# Construction and Performance Evaluation of an Astaxanthin–Chitosan/Chitooligosaccharide Hydrogel System for Ex Vivo Culture of Murine Spermatogonial Stem Cells

**DOI:** 10.3390/biology14121664

**Published:** 2025-11-24

**Authors:** Jiang Wu, Siqi Liu, Xiaowen Zeng, Yang Li, Yinlin Yao, Jing Wang, Guangdong Hu, Kai Kang

**Affiliations:** 1 College of Coastal Agricultural Sciences, Guangdong Ocean University, Zhanjiang 524088, China; wuj@gdou.edu.cn (J.W.);; 2 College of Animal Science and Technology, Shihezi University, Shihezi 832000, China

**Keywords:** 3D hydrogel, spermatogonial stem cells, transcriptomics, chitosan/chitosan oligosaccharide, astaxanthin

## Abstract

Spermatogonial stem cells (SSCs) are the only adult male germline stem cells capable of lifelong self-renewal and differentiation into spermatozoa. Scalable ex vivo survival is essential for endangered species germplasm banking, genetic resource conservation, and male infertility therapy. Here, chitosan (CO) or chitosan oligosaccharide (COS) was cross-linked into injectable, biodegradable 3D hydrogels loaded with the natural astaxanthin (AST). CCK-8 optimization identified 0.3% CO + 0.2% AST (CHAG) and 0.2% COS + 0.2% AST (COAG) as superior formulations. After 7 or 14 d of 3D culture, CHAG yielded significantly more colonies than controls (*p* < 0.01), with elevated EdU incorporation, alkaline phosphatase activity, and positive OCT4 and PLZF staining, confirming preserved stemness. Caspase-3 expression was markedly reduced, indicating the AST-mediated suppression of oxidative apoptosis. RNA-seq showed distinct transcriptome pathways (*p* < 0.01): CHAG up-regulated adhesion and ECM–receptor and cell cycle pathways, whereas COAG enriched immune-modulatory and signaling modules, enabling context-specific use. AST-loaded CO/COS hydrogels are inexpensive, cytocompatible, and scalable, doubling as a biomimetic niche that accelerates SSC proliferation while delaying senescence. The platform provides a robust, controllable 3D system for SSC expansion and establishes a pre-clinical basis for translating CO/COS/AST composites to reproductive stem cell biotechnology.

## Simple Summary

Male infertility and the loss of genetic diversity in endangered animals are urgent problems, because once spermatogonial stem cells (SSCs) are damaged or die, they cannot grow back on their own. This study aimed to create a soft, safe, and low-cost “nursery” that can keep these precious cells alive and multiplying outside the body. We molded two gentle gels from shrimp shell sugar and astaxanthin (a powerful antioxidant). After one or more weeks in these gels, murine SSCs formed far more healthy colonies than in ordinary plastic dishes; they stayed young, kept their identity, and showed almost no death signals. Gene tests showed that one gel boosts cell-to-cell glue and growth controls, while the other calms harmful inflammation. Both gels are cheap, easy to prepare, and fully biodegradable.

## 1. Introduction

Spermatogonial stem cells (SSCs) are anchored to the basement membrane of the seminiferous tubules and represent the only unipotent adult stem cell population in the male germline capable of simultaneously maintaining lifelong self-renewal and differentiation [1]. This unique property enables SSCs to continuously generate sperm throughout an individual’s lifetime, ensure the stable transmission of genetic material, and underscore their critical role in maintaining male fertility [2]. Located within the seminiferous epithelium, SSCs undergo self-renewal while also undergoing terminal differentiation into spermatozoa, thereby providing a highly tractable experimental model for the study of spermatogenesis, meiotic regulation, and cellular reprogramming [3]. However, as age progresses—and particularly during in vitro culture—SSCs are prone to cellular senescence, characterized by impaired proliferative capacity and the loss of stemness; this significantly limits their experimental and clinical utility [4].

In the adult testis, these rare SSCs reside within a specialized niche composed of Sertoli cells, peritubular myoid cells, extracellular matrix, and local soluble factors [1,5]. By supplying external cues, this microenvironment precisely balances self-renewal and differentiation, and it is indispensable for sustaining stem cell potential [6]. In conventional 2D culture, cells are seeded on flat dishes, flasks, or microfluidic chips and expand as a monolayer. This system is widely used because it is simple, reproducible, cheap, and cost-effective. Yet it allows only lateral diffusion and lacks three-dimensional complexity [7]. Three-dimensional cultures that recreate the in vivo microenvironment have gained interest. These systems offer an artificial 3D space where cells can migrate, grow, and keep the physical and structural features found in vivo [8,9]. They faithfully re-create both cell–cell and cell–extracellular matrix contacts [10,11]. Such models build these interactions to mirror the physical, nutrient, and metabolic conditions cells experience inside the body [11,12].

The reliable identification and functional upkeep of SSCs rest on a tiered set of molecular markers. For core pluripotency transcription factors, OCT4 (encoded by POU5F1 at 6p21.3) maintains embryonic and germline stem cell pluripotency through the coordinated action of its N-terminal region, POU DNA-binding domain, and C-terminal transactivation domain [13,14]; MYC boosts mouse SSC self-renewal by up-regulating glycolysis [15]; and NANOG, first isolated from blastocyst inner cell mass in 2003, has since been shown to sustain stemness in vitro [16]. SSC-specific markers include the GDNF receptor GFRα1, whose self-renewal signal is well validated [17]; PLZF (promyelocytic leukemia zinc finger), whose loss or mutation depletes germ cells [18]; and VASA (DDX4), a DEAD-box protein confirmed as a universal germ cell marker in cattle, pigs, rhesus monkeys, goats, and other mammals [19,20]. In addition, PCNA and KI67, respectively, serving as the auxiliary factor of DNA polymerase-δ and the essential protein for cell proliferation, are used to quantitatively assess proliferative activity [21,22]. Caspase 3, P21 (CDKN1A), and P16 (CDKN2A) mark apoptosis execution, G1/S-G2/M arrest, and the onset and maintenance of cellular senescence [23,24,25]. Together these core pluripotency, SSC-specific, and proliferation-to-senescence genes form an integrated molecular framework for the in vitro identification and study of SSCs.

Astaxanthin (AST) is a naturally occurring xanthophyll carotenoid found in abundance in red-hued seafood such as algae, crustaceans, and salmon [24]. Its free radical-scavenging capacity surpasses that of synthetic antioxidants, and its antioxidant activity is 100–500 times that of α-tocopherol and 15 times that of other carotenoids [26]. By restoring the redox balance, AST protects sperm mitochondrial function, preserves membrane integrity, and boosts motility [27]. In scaffolds, AST-loaded composite hydrogels also enhance fibroblast adhesion, proliferation, and VEGF expression [26,28]. Chitosan (CO), a deacetylated derivative of crustacean chitin, is degraded by lysosomal enzymes, is non-toxic and non-immunogenic, and offers excellent biocompatibility [29]. Studies show that CO-based 3D scaffolds markedly increase the proliferation of mouse MSCs (Mesenchymal stem cells) and SSCs [30,31] and concurrently improve semen quality [32]. Chitooligosaccharide (COS), an alkaline amino-oligosaccharide second only to cellulose in natural abundance, is composed of 2–10 monosaccharides linked by glycosidic bonds [33]. It markedly increases the survival of ovarian germline stem cells (OGSCs) in chemo-treated mice and drives their proliferation by up-regulating IL-2 (interleukin-2) and TNF-α (tumor necrosis factor-α), while down-regulating IL-10 (interleukin-10) and TGF-β (transforming growth factor-β) [34,35]. Together, AST, CO, and COS combine antioxidant, anti-inflammatory, pro-proliferative, and anti-senescence activities, which could provide both the theoretical rationale and the material foundation for establishing a high-efficiency 3D culture system for SSCs.

In this study, chitosan (CO), chitooligosaccharide (COS), and astaxanthin (AST) were assembled in 12 weight ratio combinations; after CCK-8 prescreening, CHAG (CO + AST) and COAG (COS + AST) hydrogels were fabricated. Multi-parallel assessments—CCK-8 proliferation curves, EdU incorporation, alkaline phosphatase activity, immunofluorescence labeling (OCT4, MYC, PLZF, VASA, Ki67, PCNA, Caspase-3, and P16), RNA-seq transcriptomes, and pathway enrichment—demonstrated that the platform sustains in vitro SSC expansion, preserves stemness, and attenuates oxidative senescence, thereby establishing its immediate utility and providing a benchmark for future investigations into the hydrogel-mediated regulation of SSC proliferation and aging.

## 2. Materials and Methods

### 2.1. Experimental Animals

In this study, 45 male Kunming mice, one-month-old in age, were housed in a specific-pathogen-free facility with ad libitum access to food and water, under a 12 h light/dark cycle, 22 ± 2 °C ambient temperature, and 50–60% relative humidity. All experimental procedures were approved by the Laboratory Animal Ethics Committee of Guangdong Ocean University (approval no. GDOU-NXY-2024-039-413).

### 2.2. Isolation and Purification of Spermatogonia

The mice were euthanized by cervical dislocation; their testes were aseptically removed after disinfection with 75% ethanol and placed in PBS containing 1% penicillin–streptomycin–ampicillin (antibiotic mixture) for transportation to the cell laboratory. The testes were rinsed 2–3 times with the same concentration of antibiotic PBS. The tunica albuginea was aseptically stripped off, and the seminiferous tubules were completely extracted. Next, 0.125% trypsin (containing 5mg/mL DNase I) was added and thoroughly pipetted. The mixture was digested at 37 °C for 9 min. Then, a double volume of DMEM/F12 (Gibco, New York, NY, USA, cat. 12500062) complete medium (containing 10% FBS, 1% non-essential amino acids, 1% sodium pyruvate, 2 mmol/L L-glutamine, and 100 U/mL penicillin–streptomycin from ExCell, Shanghai, China) was added to terminate the reaction. The mixture was centrifuged at 120× *g* for 5 min, and the supernatant was discarded. The filtrate was filtered through a 70 μm cell sieve. The filtrate was centrifuged at 200× *g* for 5 min, and the precipitate was a single-cell suspension. The cells were resuspended in complete medium, and 10 μL was stained with trypan blue for counting. The remaining suspension was inoculated into a 100 mm culture dish and cultured in a 37 °C, 5% CO_2_ incubator for 18 h for differential adhesion. The non-adherent cells were gently pipetted and collected and centrifuged at 150× *g* for 5 min. The precipitate was the primary SSCs, which were used for subsequent experiments.

### 2.3. Preparation of the Hydrogel Layer

#### 2.3.1. AST + CO Hydrogel (COnAx, 0 ≤ *n* ≤ 3, 0 ≤ x ≤ 2)

Chitosan (CO, deacetylation degree ≥ 90%, MACKLIN, Shanghai, China, cat. C909112) powder was accurately weighed and dissolved in DMEM/F12 complete medium by magnetic stirring overnight to prepare 0.1%, 0.2%, and 0.3% (*w*/*v*) stock solutions. Separately, astaxanthin (AST, Solarbio, Beijing, China, cat. SA8730) powder was dissolved in dimethyl sulfoxide (DMSO) to obtain 0.1% and 0.2% (*w*/*v*) stock solutions. The CO and AST stock solutions were then mixed at a 1:1 volume ratio (Table 1), vortexed thoroughly, and allowed to stand at room temperature for 30 min to form the COAST hydrogel. The final product was stored at 4 °C in the dark for subsequent use.

#### 2.3.2. AST + COS Hydrogel (COS nAx, 0 ≤ *n* ≤ 3, 0 ≤ x ≤ 2)

Chitosan oligosaccharide (COS, Mw < 3 kDa, MACKLIN, Shanghai, China, cat. C875644) was substituted for CO at the same mass concentrations; all subsequent steps were performed as described in Table 1. An exact 400 μL aliquot of the hydrogel was evenly layered onto the bottom of each well of a 24-well plate and allowed to gel for 2 h at 37 °C. The non-polymerized solution was aspirated, and the gel was sterilized under UV light in a laminar flow hood for 30 min. Immediately before use, each gel was rinsed twice with sterile PBS.

### 2.4. Three-Dimensional Hydrogel Cultures of Mouse Spermatogonial Stem Cells

Primary SSCs were seeded at 5 × 10^4^ cells/well onto CHAG and COAG surfaces (CHAG and COAG as shown in Table 2); wells without hydrogel served as blank controls (CG). The culture medium consisted of DMEM/F12 + 10% FBS + 20 ng/mL GDNF + 10 ng/mL bFGF + 1% triple antibiotic. Cells were maintained at 37 °C, 5% CO_2_, and 95% humidity for 7 days with half-medium exchanges every 48 h.

### 2.5. Assessment of Mouse Spermatogonial Stem Cell Proliferation

#### 2.5.1. EdU-Based Proliferation Assay

Following the Ribobio EdU-488 kit protocol (Beyotime, Nanjing, China. cat. C0075S), cells were incubated with a 2× EdU working solution (10 μmol/L) diluted 1:1 in fresh medium (final 5 μmol/L) for 2 h at 37 °C. Thereafter, cells were fixed in 4% paraformaldehyde (15 min), permeabilized with 0.3% Triton X-100 (10 min), and incubated with the Click reaction cocktail in the dark (30 min). Following nuclear counterstaining with Hoechst 33,342 (5 μg/mL), images were taken with a fluorescence microscope, and the EdU^+^/Hoechst^+^ ratio was quantified using ImageJ (version 1.53k, NIH, Bethesda, MD, USA).

#### 2.5.2. CCK-8 Viability Assay

SSCs were seeded at 1 × 10^4^ cells/well in 96-well plates (6 replicate wells per group). Then, 4 h before the end of the culture period, 10 μL CCK-8 reagents was added and incubation continued for 2 h. Absorbance at 450 nm (A450) was recorded on a microplate reader; values were blank-corrected and used to calculate the relative proliferation rate (ZETA, Sierra Madre, CA, USA, cat. K009).

### 2.6. Scanning Electron Microscopy (SEM)

The 7d cell samples were fixed overnight at 4 °C in 2.5% glutaraldehyde-0.1 mol/L PBS (pH 7.4); washed with 0.1 mol/L PBS for 3 × 15 min, and then post-fixed with 1% osmium acid for 1 h; dehydrated with ethanol gradients (30–100%) for 15 min each, and 100% ethanol was repeated twice; critical point dried, fixed with conductive carbon glue, and sprayed with gold for 30 s by an ion sputtering instrument; and observed and imaged using 15 kV field emission SEM.

### 2.7. Alkaline Phosphatase (AP) Staining

The cells were fixed with 4% paraformaldehyde for 30 min, washed with PBS for 5 × 5 min, incubated with BCIP/NBT working solution at room temperature in the dark for 30 min, terminated with distilled water, counterstained with neutral red for 5 min, and the AP^+^ clones were counted and photographed under an inverted microscope (Beyotime, Nanjing, China. cat. C3250S).

### 2.8. Indirect Immunofluorescence Microscopy

The cells samples after day 7 were fixed with 4% paraformaldehyde for 30 min, permeabilized with 0.1% Triton X-100 for 10 min, and blocked with 1% BSA for 30 min. The samples were then incubated with primary antibodies (OCT4, c-MYC, PLZF, VASA, PCNA, Ki67, Caspase-3, P16; Boster, Beijing, China, cat. MA00174, BM0238, PB0199, A02448, BM1592, PB9026, M00125-3, and PB9188, dilution 1:200) for 45 min at 37 °C, followed by corresponding Alexa Fluor 488- or 555-conjugated secondary antibodies (Boster, Beijing, China, cat. BA1127 and BA1141, dilution 1:500) for 30 min at 37 °C. After thorough washing with PBS, cell nuclei were stained with Hoechst 33,342 (Solarbio, Beijing, China, cat. CA1120) for 3 min. Finally, samples were incubated in PBS containing an antifade reagent, examined under an IX53 fluorescence microscope (Olympus, Tokyo, Japan), and the mean fluorescence intensity of cell clones was quantified using ImageJ software (version 1.53k, NIH, USA).

### 2.9. Sequencing Analysis

Cell clones from the three groups (CHAG, COAG, and CG) were harvested in −80 r, and three biological replicates per group were collected for subsequent sequencing analysis.

Total RNA, isolated with TRIzol and verified for integrity (RIN ≥ 7.0) on an Agilent 2100 Bioanalyzer (Santa Clara, CA, USA), was used for library preparation. Poly (A) +mRNA was captured with oligo(dT) beads, fragmented, and reverse-transcribed into double-stranded cDNA. Following end repair, A-tailing, and adapter ligation, the libraries were PCR-amplified, quantified (Qubit), and validated (qPCR). Sequencing was performed on an Illumina NovaSeq 6000 platform (San Diego, CA, USA) (2 × 150 bp) to yield ≥ 6 Gb of high-quality (Q30 ≥ 90%) clean data per sample.

Gene-level abundances were quantified as FPKM with RSEM. Differential expression was assessed by edgeR v3.32.1, adopting thresholds of FDR < 0.05 and |log_2_FC| > 1. GO and KEGG enrichment analyses were performed with clusterProfiler v3.14.3, considering q < 0.05 as significant. Global expression patterns were inspected using principal component analysis (PCA).

### 2.10. Statistical Analyses of Experimental Data

Data are presented as mean ± SD with *n* ≥ 3. Two-group comparisons were performed using two-tailed Student’s *t*-tests; multiple-group comparisons were analyzed by one-way ANOVA followed by Tukey’s post hoc test. *p* < 0.05 was considered statistically significant and *p* < 0.01 highly significant. Graphs were generated with GraphPad Prism (version 9.0, GraphPad Software, Inc., La Jolla, CA, USA).

## 3. Results

### 3.1. Hydrogel Fabrication and Its Impact on Cellular Viability

**Figure 1:**
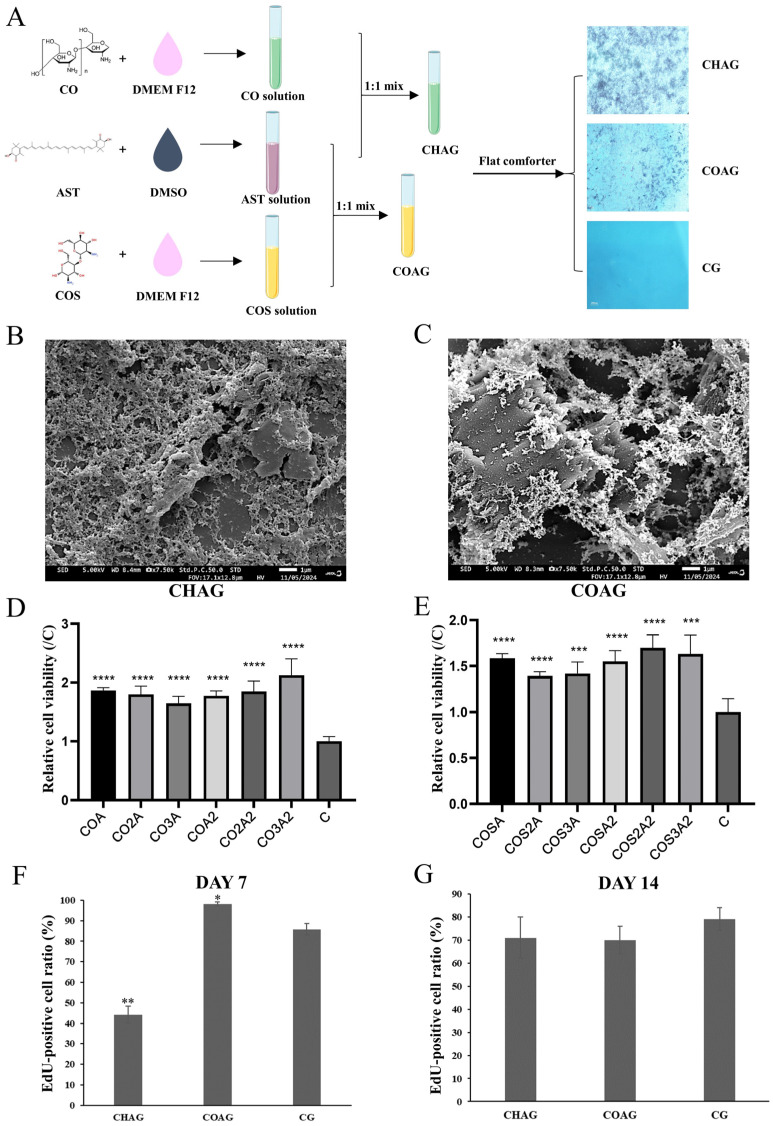
Preparation pipeline and quality assessment of AST-loaded CO/COS hydrogels. (**A**) Process diagram: dissolution of AST and CO/COS → equal volume mixing → gelation at 37 °C → UV sterilization → AST-loaded CO/COS hydrogels. (**B**,**C**) SEM micrographs of the hydrogels: B depicts the CHAG group, and C depicts the COAG group; both reveal a porous, three-dimensional architecture (white scale bar = 1 µm, in the lower-right corner of the image). (**D**,**E**) Cell viability: CCK-8 screening of 12 formulations after 1 day; *** *p* < 0.001, **** *p* < 0.0001 vs. C, identifying 0.2% AST + 0.3% CO (CHAG) and 0.2% AST + 0.2% COS (COAG) as optimal (*n* = 5). (**F**,**G**) EdU proliferation: representative images and quantification of EdU^+^ SSCs on CHAG, COAG, and CG at day 7 and 14; proliferation at day 7 is significantly different from CG (* *p* < 0.05, ** *p* < 0.01 vs. CG) (*n* = 5).

Composite hydrogels were prepared from astaxanthin (AST), chitosan (CO), and chitooligosaccharide (COS) powders. Light microscopy revealed a homogeneous, sheet-like distribution of the gel (Figure 1A). Scanning electron microscopy (SEM) further disclosed a loose, highly porous 3D network with pore diameters conducive to cell adhesion and nutrient diffusion (Figure 1B,C).

To evaluate the effects of different hydrogel formulations on the viability of spermatogonial stem cells (SSCs), a CCK-8 assay was performed on culture day 3 (the morphology of SSCs in the CHAG, COAG, and CG groups are shown in Figure S1) to screen 12 concentration combinations (as shown in Table 1). The results showed that the absorbance at 450 nm was significantly higher in the 0.2% AST + 0.3% CO and 0.2% AST + 0.2% COS groups compared with all other ratios (*p* < 0.01; Figure 1D,E), indicating their superior pro-proliferative effects on SSCs. Based on these findings, these two optimized formulations were selected for all subsequent experiments and designated as CHAG (0.2% AST + 0.3% CO) and COAG (0.2% AST + 0.2% COS), respectively.

SSCs were inoculated onto the surfaces of CHAG and COAG and continuously cultured for 7 days and 14 days (as shown in Figure S1), followed by EdU incorporation experiments to compare the proliferation kinetics. The results showed that in the CHAG group, the proportion of EdU^+^ cells on day 7 was significantly lower than that in the control (CG) group (*p* < 0.01), but rebounded by day 14 to a level not statistically different from CG (Figure 1F); conversely, in the COAG group, the EdU^+^ fraction on day 7 was significantly higher than in CG (*p* < 0.05), yet declined by day 14 to match CG levels (Figure 1G), suggesting that proliferation fatigue might occur with long-term culture. To ensure that the detection window was within the exponential proliferation phase, all subsequent functional evaluations were conducted at the 7-day time point.

### 3.2. Isolation, Culture, and Phenotypic Characterization of SSCs

**Figure 2:**
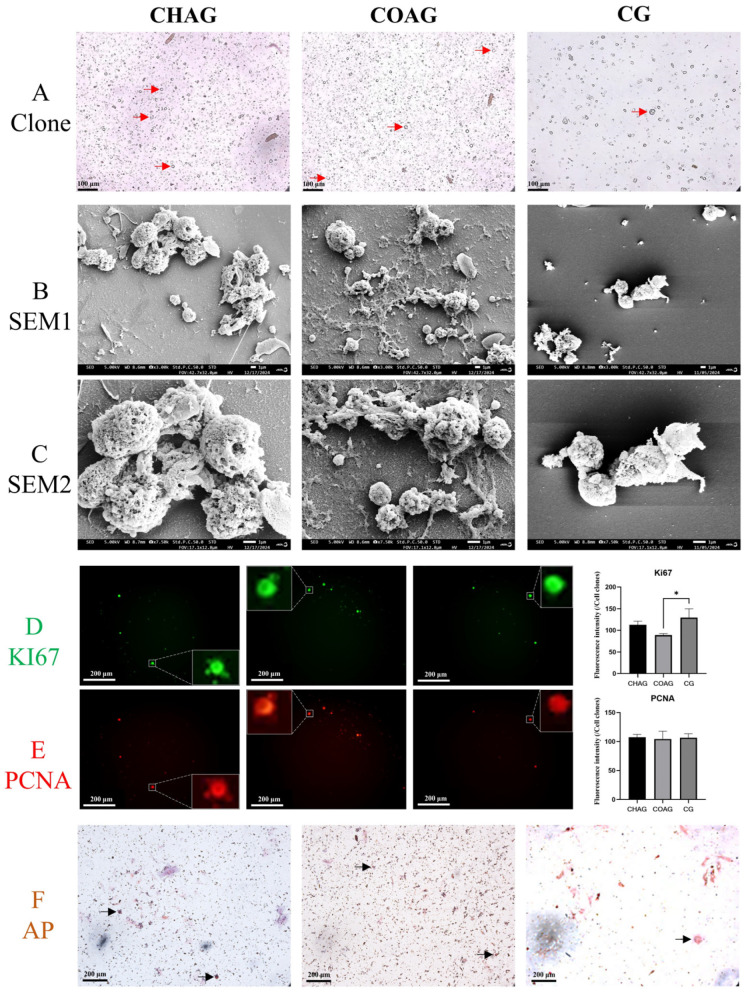
Morphology, clonogenicity, and functional assessment of mouse SSCs cultured for 7 days on AST-loaded CO/COS hydrogels. (**A**) Clone line: bright-field observation of SSC clones after 7 days of culture (cell clones are indicated by the red arrows; white scale bar (=100 μm) is shown in the lower-left corner of the image); clone counts in individual wells of 6-well plates (34 mm diameter, ~9 cm^2^ area) were as follows: CHAG 738.8 ± 148.9, COAG 508.6 ± 74.8, CG 466.5 ± 63.0; *p* < 0.01 vs. CG (*n* = 3). (**B**,**C**) SEM1 and SEM2 lines: scanning electron microscopy of SSCs after 7 days of culture (white scale bar = 1 μm, in the lower-right corner of the image). (**D**,**E**) KI67 and PCNA lines: immunofluorescence staining for proliferation-related genes (Ki67 and PCNA) after 7 days of SSC culture (white scale bar = 200 μm, in the lower-left corner of the image), with mean fluorescence intensity of each group compared to CG (clone number *n* = 20, * *p* < 0.05). (**F**) AP line: alkaline phosphatase staining of SSCs after 7 days of culture (cell clones are indicated by the blank arrows; white scale bar = 200 μm, in the lower-left corner of the image).

After 7 days of culture, SSC colonies were examined. CHAG exhibited dense cell clusters; COAG showed a moderate decrease in cluster density; and CG displayed sparse cells, mostly single (Figure 2A). Clone numbers were quantified by ImageJ analysis of independent wells (six-well plates, 34 mm diameter, ~9 cm^2^ area): CHAG 738.8 ± 148.9, COAG 508.6 ± 74.8, and CG 466.5 ± 63.0 (CHAG and COAG vs. CG, *p* < 0.01). Scanning electron microscopy images (Figure 2B,C) showed distinct growth patterns: CHAG cells aggregated into large clonal clusters with high cell numbers; COAG had moderate cell numbers with less pronounced aggregation; and CG cells were sparse and showed no cluster formation.

Immunofluorescence further confirmed that Ki67 and PCNA were expressed in both CHAG and COAG. Except for Ki67 in COAG being significantly lower than in CG (*p* < 0.05), the mean fluorescence intensities of other groups showed no significant difference versus CG (Figure 2D,E), indicating that AST-loaded CO/COS hydrogels maintain SSC survival in vitro. Stem cell identity was assessed by alkaline phosphatase (AP) activity staining: AP staining revealed strong orange-red deposits in CHAG and COAG groups, whereas the signal in CG was faint (Figure 2F).

### 3.3. Immunofluorescence Assessment of SSC Identity, Stemness Maintenance, and Senescence Markers

**Figure 3:**
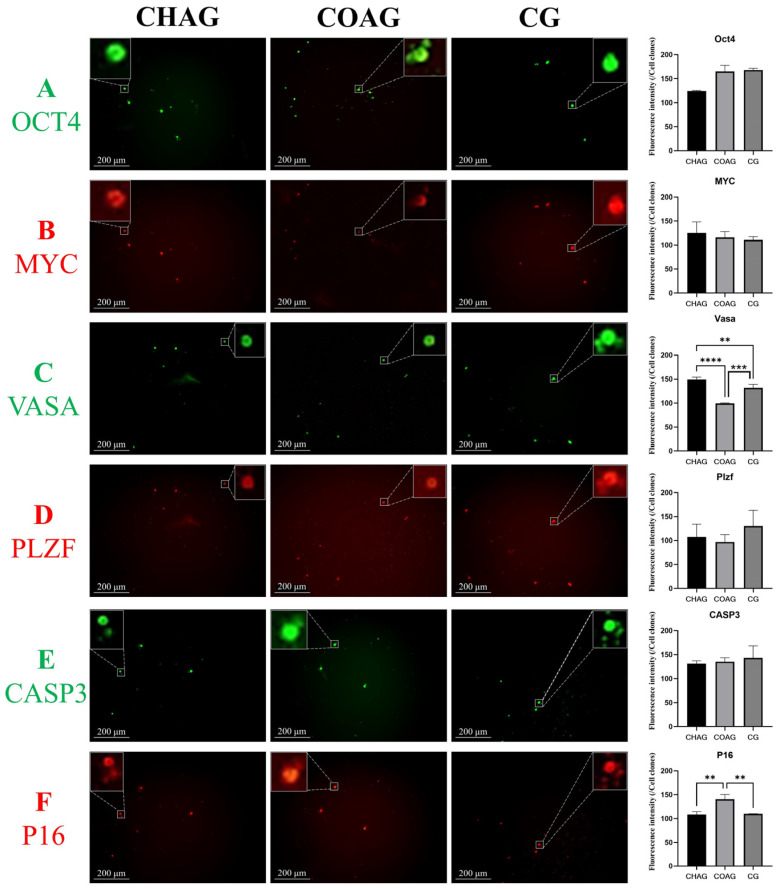
Immunofluorescence profiling of molecular markers in mouse SSCs after 7 days on AST-CO/COS hydrogels. (**A**,**B**) OCT4 and MYC lines, immunofluorescence co-staining (white scale bar = 200 μm, in the lower-left corner of the image), and quantitative analysis of mean fluorescence intensity for stemness transcription factors OCT4 and MYC in CHAG, COAG, and CG groups (clone number = 20). (**C**,**D**) VASA and PLZF lines, immunofluorescence co-staining (white scale bar = 200 μm, in the lower-left corner of the image), and quantitative analysis of mean fluorescence intensity for SSC-specific markers VASA and PLZF in CHAG, COAG, and CG groups (clone number = 20, ** *p* < 0.01, *** *p* < 0.001, **** *p* < 0.0001). (**E**,**F**) CASP3 and P16 lines, immunofluorescence co-staining (white scale bar = 200 μm, in the lower-left corner of the image) and quantitative analysis of mean fluorescence intensity for senescence-related genes Caspase-3 and P16 in CHAG, COAG, and CG groups (clone number = 20, ** *p* < 0.01).

To verify whether CHAG and COAG hydrogels retain the molecular identity of SSCs, cells cultured for 7 days were co-stained for OCT4 and MYC (two SSC stemness markers) and for VASA and PLZF (two SSC germ cell-specific markers). Immunofluorescence analysis (Figure 3A,B) showed that OCT4 and MYC were expressed in both CHAG and COAG groups. Mean fluorescence intensity comparisons revealed no significant difference versus CG. Specifically, OCT4 intensity in CHAG was lower than in CG, while MYC was higher; in COAG, both genes were slightly higher than CG without statistical significance.

As shown in Figure 3C,D, VASA intensity in CHAG was markedly higher than in CG (*p* < 0.01), whereas PLZF was slightly lower without significance. In COAG, VASA intensity was particularly lower than CG (*p* < 0.001), and PLZF was also slightly lower without significance. These results indicate that the SSC-specific phenotypic profile is preserved, confirming that the 3D hydrogel platform effectively maintains core SSC marker genes.

For cellular senescence, Caspase-3 (early apoptosis executor) and P16 (senescence-related cell cycle arrest factor) were analyzed using immunofluorescence (Figure 3E,F). Caspase-3 intensity in CHAG was slightly lower than CG without significance, and P16 was also slightly lower without significance. In COAG, Caspase-3 intensity was comparable to CG without significance, while P16 was significantly higher than CG (*p* < 0.01). This suggests that AST-loaded CO/COS hydrogels delay in vitro SSC senescence by inhibiting apoptosis and senescence pathways.

In summary, CHAG and COAG not only retain SSC molecular identity and stemness but also confer anti-senescence capacity.

### 3.4. Comparative mRNA Profiling of SSCs Cultured Under Distinct Hydrogel Systems

**Figure 4:**
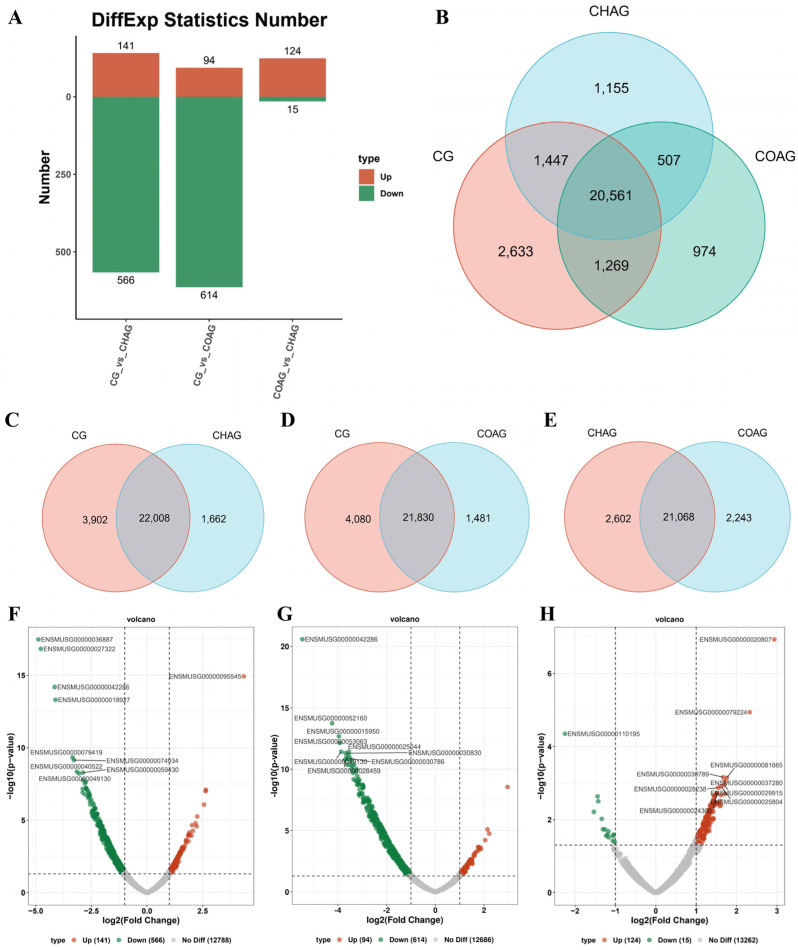
Multi-platform comparative transcriptome landscape and core differential genes. (**A**) Differential expression statistics: number of up-regulated (red) and down-regulated (green) genes for each comparison. (**B**) Three-way Venn diagram displaying total, unique, and overlapping gene counts among CHAG, COAG, and CG groups. (**C**) Venn diagram: unique and shared differential genes between CHAG and CG. (**D**) Venn diagram: unique and shared differential genes between COAG and CG. (**E**) Venn diagram: unique and shared differential genes between CHAG and COAG. (**F**) Volcano plot of DEGs in CHAG vs. CG: red, up-regulated; green, down-regulated. (**G**) Volcano plot of DEGs in COAG vs. CG: red, up-regulated; green, down-regulated. (**H**) Volcano plot of DEGs in CHAG vs. COAG: red, up-regulated; green, down-regulated.

The comprehensive analysis of whole-cell mRNA transcriptome sequencing revealed that hundreds of genes exhibited up- or down-regulation (Figure 4A), along with a considerable number of genes showing the elimination of intergroup expression differences among CHAG, COAG, and CG (Figure 4B).

In CHAG vs. CG, the Venn diagram analysis (Figure 4C) further indicated that there were 22,008 genes commonly expressed in both the CHAG and CG groups, among which 1662 genes were not detected in the CG group. The results of transcriptome sequencing showed that through the volcano plot (Figure 4F) and the table of differentially expressed genes (Table S1), it was observed that compared with the CG group, the expression of genes such as Zfp969, Saa3, and Serpina3h in SSCs on CHAG was significantly up-regulated (*p* < 0.01), while the expression of genes such as C1qa, Siglec1, Stab1, Ccl6, and Ms4a6c was significantly down-regulated (*p* < 0.01). This suggested that CHAG treatment exerted a significant regulatory effect on the gene expression of mouse SSCs, inducing a unique expression profile.

In COAG vs. CG, the Venn diagram analysis (Figure 4D) revealed that 21,830 genes were expressed consistently between the COAG and CG groups, while 1481 genes were unique to the COAG group and were not detected in the CG group, further highlighting the similarities and differences in gene expression between the two groups. Similarly, as shown in the volcano plot (Figure 4G) and the table of differentially expressed genes (Table S2), in the comparison between the COAG group and the CG group, the expression levels of genes such as Stab1, Pld4, Ncf1, Clec12a, and C5ar1 significantly decreased (*p* < 0.01), while the expression levels of genes such as Saa3, Serpina3h, Angptl7, Insl3, and Ism1 significantly increased (*p* < 0.01).

In CHAG vs. COAG, the Venn diagram analysis (Figure 4E) showed that there were 21,068 genes commonly expressed in both the CHAG and COAG groups, among which 2602 genes were expressed only in the CHAG group and 2243 genes were expressed only in the COAG group; this indicated that both gel systems induced unique gene expression profiles at the transcriptional level. Further analysis through the volcano plot (Figure 4H) and the table of differentially expressed genes (Table S3) revealed that in the direct comparison between the CHAG group and the COAG group, the expression levels of genes such as Pde2a, Zfp968, Rps13-ps2, Gm47739, and Gm15429 were significantly down-regulated (*p* < 0.01), while the expression levels of genes such as 4933427D14Rik, Gm6565, Itgax, Pira1, and Galnt6 were significantly up-regulated (*p* < 0.01).

### 3.5. Pathway-Centric Clustering Analysis of Differentially Expressed Genes

In CHAG vs. CG, the KEGG analysis revealed that compared with the CG group, the pathways enriched in CHAG (relative to CG) under the “Cellular Processes” category included focal adhesion, cell cycle, and tight junction (all *p* < 0.01), while under “Environmental Information Processing,” the cell adhesion molecule (CAM) pathway was also significantly enriched (*p* < 0.01) (Table 3, Figure S2). GO functional annotation further showed that at the cellular component level, CHAG-cultured SSCs were significantly enriched in terms including the cell periphery, cell surface, extracellular matrix, external encapsulating structure, protein complexes involved in cell adhesion, plasma membrane, and collagen-containing extracellular matrix (all *p* < 0.01); at the molecular function level, the binding activities of cell adhesion molecules were markedly enhanced (*p* < 0.01); and at the biological process level, significant enrichment was observed in responses to external stimuli, cell migration, cell adhesion, cell motility, and the regulation of multicellular organismal processes (all *p* < 0.01) (Figure S3).

In COAG vs. CG, the KEGG analysis revealed that, compared with the CG group, the COAG group exhibited significant enrichment in several pathways. Under “Cellular Processes”, focal adhesion and regulation of the actin cytoskeleton were enriched (*p* < 0.01). Within “Environmental Information Processing,” the MAPK signaling pathway and ECM–receptor interaction pathway were significantly over-represented (*p* < 0.01). In the “Organismal Systems” category, enriched pathways included osteoclast differentiation, chemokine signaling, leukocyte transendothelial migration, B cell receptor signaling, platelet activation, C-type lectin receptor signaling, complement and coagulation cascades, and Fc-γ-R-mediated phagocytosis (all *p* < 0.01) (Table 4, Figure S4). GO analysis indicated significant enrichment at the biological process level for cell adhesion, at the cellular component level for structures such as the cell periphery, cell surface, plasma membrane, extracellular matrix, and anchoring junction, and at the molecular function level for cell adhesion molecule binding activity (all *p* < 0.01) (Figure S5).

In CHAG vs. COAG, the KEGG analysis showed that, compared with COAG, CHAG was significantly enriched in phagosome and actin cytoskeleton regulation under “Cellular Processes” (*p* < 0.01). In “Environmental Information Processing,” the cytokine–cytokine receptor interaction pathway was markedly over-represented (*p* < 0.01). Under “Organismal Systems,” CHAG exhibited significant enrichment in pathways including osteoclast differentiation, B cell receptor signaling, natural killer cell-mediated cytotoxicity, leukocyte transendothelial migration, neutrophil extracellular trap formation, C-type lectin receptor signaling, FcγR-mediated phagocytosis, and complement and coagulation cascades (all *p* < 0.01) (Table 5, Figure S6). GO annotation further indicated that, at the biological process level, CHAG was enriched in immune-related processes such as immune response, immune system regulation, cell activation, defense response, and leukocyte activation (*p* < 0.01). At the cellular component level, enrichment was observed in membrane-related structures including the plasma membrane, cell periphery, cell surface, and integral membrane components (*p* < 0.01). At the molecular function level, CHAG showed enhanced activity in immune receptor binding, pattern recognition, MHC class I activity, cell adhesion molecule binding, and superoxide-generating NADPH oxidase activation (*p* < 0.01) (Figure S7).

## 4. Discussion

In this study, we successfully fabricated two composite hydrogels, designated CHAG and COAG. Light and scanning electron microscopy showed that both hydrogels exhibited a sheet-like morphology with a loose, highly porous internal structure (Figure 2). This perforated three-dimensional network provided ample anchoring sites for cells, while enabling efficient nutrient diffusion and waste metabolite removal, thereby supporting SSC proliferation and functional maintenance [1,5]. The screening of hydrogel formulations by CCK-8 assay identified 0.3% CO + 0.2% AST (CHAG) and 0.2% COS + 0.2% AST (COAG) as the optimal hydrogel ratios for promoting murine SSC ex vivo culture. Notably, proliferative activity was significantly higher at day 7 than at day 14, suggesting that extended culture leads to gradual senescence and a corresponding decline in replicative capacity.

Hydrogels are three-dimensional networks composed of cross-linked natural or synthetic polymer chains [36]. Naturally derived hydrogels are widely used as effective 3D cell culture matrices due to their high-water content and physicochemical/biological similarities to native tissues [37]. They can be applied alone or combined with biological scaffolds, basement membrane extracts, or microfluidic devices to meet the specific needs of different cell types [38,39]. These natural hydrogels contain abundant extracellular matrix (ECM) components and bioactive factors—such as chitooligosaccharides, collagen, and laminin [40,41,42]—which support cell survival, proliferation, differentiation, and function [43,44]. Additionally, their inherent biocompatibility and bioactivity help create a favorable microenvironment that promotes optimal cellular performance. In this study, compared with existing murine SSC culture systems, the AST-CO/COS hydrogel outperformed the chitosan/graphene oxide nanocomposite (CA-GO) in “safety–biodegradability–antioxidation” [31] and surpassed pure chitosan film in mechanical matching and immune quiescence [45]. It also possessed component-defined and injectable/printable advantages absent in a decellularized testicular matrix (DTM) [46]. Although the 7-day expansion fold was slightly lower than CA-GO and DTM [31,45], the integrated indices of expansion efficiency, cost, and regulatory precision indicated significant superiority in antioxidant capacity, cost control, and operational convenience over existing reports, while long-term in vivo function still awaited final validation through transplantation germline transmission experiments. Innovative hydrogel technologies could further enhance application potential [5,47]: Janus hydrogels exhibited remarkable advantages in mimicking extracellular matrix heterogeneity, modulating cellular behavior, and promoting tissue regeneration [47]; this could represent a key direction for future innovation in this study.

Astaxanthin (AST) is a naturally occurring xanthophyll carotenoid abundant in algae, crustaceans, and salmonid fish [48]. Liu et al. reported that AST restored the oxidant–antioxidant balance within the male reproductive system, preserved mitochondrial function in spermatozoa, and improved semen quality and attributable effects, at least in part, to reduce oxidative damage, maintain membrane integrity, and enhance sperm motility parameters [49]. Choi et al. supplemented astaxanthin (AST) into gelatin–methacryloyl (GelMA) hydrogels and observed a marked enhancement in mesenchymal stem cell (MSC) proliferation [28]. Similarly, Zhang et al. encapsulated MSCs in AST-loaded methoxy-poly (ethylene glycol)-b-polycaprolactone polymeric micelles and reported that 0.5 μg/mL AST increased cell numbers by 26.3% within 8d [45]. Monavari et al. reported that AST-incorporated composite hydrogels enhance fibroblast adhesion, proliferation, and VEGF expression [26], while Afzali et al. demonstrated that AST mitigates busulfan-induced oxidative apoptosis in human SSCs via the activation of the Nrf-2/HO-1 pathway [27].

Chitosan (CO), the deacetylated derivative of chitin, was non-toxic and non-immunogenic, and its degradation products elicited no adverse systemic reactions [50]. Owing to its excellent biocompatibility and biodegradability, CO was regarded as a safe biomaterial for biomedical applications [51,52]. Previous studies had shown that CO could be combined with various polymers to generate three-dimensional scaffolds suitable for cell culture [29]. Uslu et al. found that simply adding soluble CO to the culture medium enhanced cell growth, behavior, and intercellular junctions, thereby improving tissue architecture [50]. Naeemi et al. reported that hyaluronic acid/CO scaffolds sustained SSC differentiation and proliferation [46]. Moeinzadeh et al. demonstrated that CO-based nanocomposite scaffolds exhibited excellent biocompatibility and promoted SSC adhesion and proliferation [31]. Similarly, Su et al. found that nano-hydroxyapatite/CO/PLGA scaffolds effectively inhibited mesenchymal stem cell senescence [53].

Chitooligosaccharide (COS), a basic amino-oligosaccharide second only to cellulose in natural abundance, consists of 2–10 monosaccharides linked by glycosidic bonds [33,54]. Huang et al. found that COS markedly improved ovarian function and immune status in chemotherapy-treated mice, which increased OGSC survival [34]. Zheng et al. reported that COS promoted the proliferation of ovarian germline stem cells (OGSCs), while up-regulating IL-2 and TNF-α and down-regulating IL-10 and TGF-β [35].

After 7 days of in vitro culture, the number of SSC clones in both the CHAG and COAG groups was significantly higher than that in the CG group, indicating that both hydrogels promote proliferation. Immunofluorescence revealed the pronounced expression of the proliferation markers Ki67 and PCNA in CHAG and COAG, further corroborating their proliferative effect. This disparity likely stems from the distinct microenvironments created by each hydrogel formulation. CO and COS exhibit excellent biocompatibility and cell adhesive properties, furnishing a stable substrate for anchorage, whereas AST mitigates oxidative stress-induced injury through antioxidant activity, collectively accelerating proliferation. Owing to the compositional differences between CHAG and COAG, their respective proliferative and anti-senescence capacities also diverge.

KEGG profiling revealed that CHAG-up-regulated genes were significantly enriched in pathways governing cell adhesion and ECM–receptor interaction, implying that the hydrogel orchestrates collective biological events including cell motility, adhesion, cell cycle progression, and extracellular matrix remodeling. GO analysis further indicated that CHAG-up-regulated genes were markedly enriched in functional terms including the extracellular matrix, cell surface, extracellular space, plasma membrane, cell periphery, protein binding, cell adhesion molecule binding, ion binding, and signaling receptor binding, suggesting that the hydrogel enhances intercellular communication, signal transduction, and extracellular environment modulation. Augustine et al. confirmed that CO–ascorbic acid hydrogel could enhance the adhesion ability of HUVEC and hMSCs [55]. Tashakkorian et al. further reported that a CO–polyvinyl alcohol hydrogel likewise promoted cellular adhesion [56]. Guan et al. documented that a CO–gelatin–nanoparticle hydrogel augmented the adhesion, proliferation, and differentiation of neural stem cells [57]. Zhao et al. showed that an in situ-forming CO-based hydrogel enhanced keratinocyte proliferation and migration [58]. Ding et al. verified that a thiolated-CO-functionalized hydrogel accelerates osteoblast proliferation and modulates Wnt signaling [59]. Liu et al. found that a CO-β-glycerophosphate-collagen-stromal-cell-laden hydrogel suppressed tenocyte apoptosis via the AKT/GSK-3β axis [60].

In the COAG group, genes were predominantly enriched in pathways related to cell adhesion and immune system regulation, implying that this hydrogel modulates biological events such as cell motility, adhesion, signal transduction, tumorigenesis and tumor progression, cardiac disorders, and immune responses. Zhai et al. reported that COS significantly restored the immune organ index, phagocytic index, lymphocyte proliferation, NK-cell activity, and antioxidant enzyme activity (*p* < 0.05) [61]. Ding et al. further demonstrated that a COS-containing hybrid bone cement up-regulated immunomodulatory factor expression in MSCs and fostered osteogenic differentiation [62].

Compared with COAG, CHAG yielded 2602 differentially expressed genes that were chiefly enriched for immune response, cytoskeleton-mediated motility, and metabolic pathways, underscoring the divergent modulation of SSC immune function, cell movement, and metabolism between the two hydrogels. For instance, CHAG-up-regulated Zfp969 might be linked to proliferation and differentiation, whereas COAG-up-regulated Saa3 and Serpina3h were likely to participate in inflammatory and immune-regulatory processes. This divergence presumably originated from compositional and structural disparities: the higher CO content in CHAG favored cell adhesion and proliferation, whereas the elevated COS fraction in COAG preferentially supported immunomodulation and anti-inflammatory responses. Moreover, the pore architecture and mechanical attributes of the two hydrogels might independently modulate cellular behavior and gene expression. The pore geometry of CHAG appears optimized for cell accommodation and mass exchange, thereby potentiating proliferation; whereas the COAG pore configuration might preferentially facilitate immunomodulatory and inflammatory signaling, consequently shaping the observed transcriptional profile.

Collectively, these data established that both CHAG and COAG serve as permissive substrates for the in vitro culture of mouse SSCs, promoting adhesion, enhancing proliferation, and attenuating cellular senescence. The absence of a head-to-head comparison with gold-standard matrices (e.g., Matrigel, collagen, or decellularized testicular matrix (DTM)) may inflate the apparent superiority reported here; therefore, our ongoing work will integrate Matrigel and DTM controls alongside detailed time–dose kinetics and in vivo transplantation to rigorously quantify the long-term maintenance, stemness retention, and germline transmission capacity of CHAG/COAG.

## 5. Conclusions

CHAG and COAG hydrogels effectively maintained murine SSC stemness, promoted proliferation, and reduced cellular senescence in vitro; this demonstrated their suitability as three-dimensional culture platforms. Transcriptomic analysis revealed distinct regulatory mechanisms: CHAG specifically up-regulated genes which were associated with cytoskeletal reorganization, cell adhesion, and extracellular matrix interactions, while COAG modulated immune response and signal transduction pathways. These differential expression profiles suggested that CHAG and COAG supported SSC survival through complementary molecular pathways. Thus, our work could establish a conceptual and experimental foundation for applying CO/COS/AST composite hydrogels in three-dimensional SSC cultivation.

## Figures and Tables

**Table 1:** AST + CO/COS gel configuration combinations.

	0.1%AST	0.2%AST
0.1%CO	0.1%CO + 0.1%AST (COA)	0.1%CO + 0.2%AST (COA2)
0.2%CO	0.2%CO + 0.1%AST (CO2A)	0.2%CO + 0.2%AST (CO2A2)
0.3%CO	0.3%CO + 0.1%AST (CO3A)	0.3%CO + 0.2%AST (CO3A2)
0.1%COS	0.1%COS + 0.1%AST (COSA)	0.1%COS + 0.2%AST (COSA2)
0.2%COS	0.2%COS + 0.1%AST (COS2A)	0.2%COS + 0.2%AST (COS2A2)
0.3%COS	0.3%COS + 0.1%AST (COS3A1)	0.3%COS + 0.2%AST (COS3A2)

**Table 2:** Mouse SSCs culture groups.

CHAG Group	COAG Group	CG Group
0.3% chitosan with 0.2% astaxanthin loading	0.2% astaxanthin-loaded 0.2% chitosan oligosaccharide	No gel

**Table 3:** KEGG pathway-centric clustering analysis of differentially expressed genes in CHAG vs. CG (*p*-value < 0.01).

Pathway ID	Pathway Description	GeneRatio	*p*-Value	Names of the Genes in Pathway
mmu04810	Regulation of actin cytoskeleton	26/370	9.94 × 10^−7^	*Actg2*, *Myh11*, *Itgal*, *Itgam*, *Itga4*, *Fn1*, *Itgb2*, *Itga6*, *Pdgfb*, *Itgax*, *Vav1*, *Cxcr4*, *Pik3cd*, *Lpar4*, *Fgf10*, *Myh10*, *Diaph3*, *Acta2*, *Itga2*, *Itga1*, *Iqgap2*, *Nckap1l*, *Itgb5*, *Scin*, *Vav3*, *Iqgap3*
mmu04510	Focal adhesion	22/370	1.10 × 10^−5^	*Actg2*, *Col6a3*, *Ccnd1*, *Itga4*, *Parvg*, *Igf1*, *Fn1*, *Lama5*, *Itga6*, *Pdgfb*, *Pgf*, *Thbs2*, *Vav1*, *Pik3cd*, *Thbs3*, *Parvb*, *Acta2*, *Itga2*, *Itga1*, *Itgb5*, *Lamc3*, *Vav3*
mmu04110	Cell cycle	15/370	0.000622	*Ccnd1*, *Tgfb3*, *Fbxo5*, *Sfn*, *Skp2*, *Pkmyt1*, *Bub1b*, *Aurkb*, *E2f2*, *Mcm5*, *Tgfb2*, *Cdc20*, *Ndc80*, *Cdc25c*, *Cdc25b*
mmu04520	Adherens junction	10/370	0.005086	*Actg2*, *Ptprb*, *Was*, *Ptpn6*, *Cdh5*, *Map3k7cl*, *Acta2*, *Iqgap2*, *Cdh1*, *Iqgap3*
mmu04512	ECM–receptor interaction	12/370	0.000166	*Col6a3*, *Itga4*, *Fn1*, *Lama5*, *Itga6*, *Thbs2*, *Thbs3*, *Npnt*, *Itga2*, *Itga1*, *Itgb5*, *Lamc3*
mmu04514	Cell adhesion molecules	23/370	0.002206	*Siglec1*, *Itgal*, *H2-T24*, *Selplg*, *Itga4*, *Cdh3*, *Evi2a*, *Sdc3*, *L1cam*, *Milr1*, *Vsir*, *Itgb2*, *Itga6*, *Slitrk4*, *F11r*, *Cdh5*, *Ptprc*, *Cd28*, *Mpzl2*, *Cntnap2*, *Cd80*, *Cdh1*, *Spn*
mmu05205	Proteoglycans in cancer	23/370	2.40 × 10^−6^	*Actg2*, *Ccnd1*, *Camk2a*, *Igf1*, *Plau*, *Fn1*, *Mmp2*, *Thbs2*, *Ptpn6*, *Vav1*, *Pik3ip1*, *Pik3cd*, *Mapk13*, *Hcls1*, *Gpc3*, *Acta2*, *Plaur*, *Itga2*, *Iqgap2*, *Itgb5*, *Tgfb2*, *Vav3*, *Iqgap3*
mmu05410	Hypertrophic cardiomyopathy	14/370	4.17 × 10^−5^	*Actg2*, *Des*, *Itga4*, *Igf1*, *Tgfb3*, *Itga6*, *Ace*, *Tmem178*, *Acta2*, *Itga2*, *Itga1*, *Mylk*, *Itgb5*, *Tgfb2*
mmu05412	Arrhythmogenic right ventricular cardiomyopathy	11/370	0.00078	*Actg2*, *Des*, *Itga4*, *Itga6*, *Tmem178*, *Jup*, *Acta2*, *Itga2*, *Itga1*, *Itgb5*, *Dsp*
mmu05032	Morphine addiction	10/370	0.001603	*Pde2a*, *Pde7b*, *Cacna1a*, *Gabra4*, *Gng2*, *Arrb2*, *Gabbr1*, *Pde3b*, *Pde1b*, *Grk3*
mmu05206	MicroRNAs in cancer	14/370	0.003315	*Ccnd1*, *Hmga2*, *Plau*, *Pdgfb*, *Thbs2*, *Pik3ip1*, *Mmp16*, *Pik3cd*, *E2f2*, *Kif23*, *Notch1*, *Tgfb2*, *Cdc25c*, *Cdc25b*
mmu05418	Fluid shear stress and atherosclerosis	13/370	0.004012	*Actg2*, *Ncf1*, *Milr1*, *Pdgfb*, *Mmp2*, *Trpv4*, *Pik3cd*, *Cdh5*, *Mapk13*, *Map3k7cl*, *Acta2*, *Ncf2*, *Il1a*
mmu05219	Bladder cancer	6/370	0.005041	*Ccnd1*, *Mmp2*, *Thbs2*, *E2f2*, *Upk3b*, *Cdh1*
mmu04380	Osteoclast differentiation	23/370	5.81 × 10^−10^	*Ncf1*, *Csf1r*, *Milr1*, *Sirpa*, *Tnfrsf11a*, *Fcgr2b*, *Pira2*, *Lcp2*, *Lilrb4a*, *Blnk*, *Pik3cd*, *Fcgr3*, *Mapk13*, *Tyrobp*, *Btk*, *Map3k7cl*, *Spi1*, *Tnfrsf11b*, *Tgfb2*, *Ncf2*, *Il1a*, *Pira12*, *Ppp3r2*
mmu04062	Chemokine signaling pathway	21/370	8.37 × 10^−6^	*Ccl6*, *Ncf1*, *Ccl9*, *Was*, *Dock2*, *Pf4*, *Cxcl1*, *Pik3cg*, *Cxcl2*, *Hck*, *Plcb2*, *Vav1*, *Cxcr4*, *Gng2*, *Arrb2*, *Prex1*, *Pik3cd*, *Lyn*, *Rasgrp2*, *Vav3*, *Grk3*
mmu04670	Leukocyte transendothelial migration	17/370	9.86 × 10^−6^	*Actg2*, *Ncf1*, *Itgal*, *Itga4*, *Milr1*, *Itgb2*, *Mmp2*, *Vav1*, *Cxcr4*, *F11r*, *Rapgef3*, *Pik3cd*, *Cdh5*, *Mapk13*, *Acta2*, *Ncf2*, *Vav3*
mmu04611	Platelet activation	16/370	3.52 × 10^−5^	*Actg2*, *Fcer1g*, *Pik3cg*, *Plcb2*, *Lcp2*, *Tbxas1*, *Pik3cd*, *Mapk13*, *Btk*, *Prkg2*, *Fermt3*, *Lyn*, *Acta2*, *Itga2*, *Apbb1ip*, *Rasgrp2*
mmu04662	B cell receptor signaling pathway	17/370	0.001055	*Cd72*, *Inpp5d*, *Milr1*, *Fcgr2b*, *Pira2*, *Ptpn6*, *Vav1*, *Lilrb4a*, *Blnk*, *Pik3cd*, *Pik3ap1*, *Fcgr3*, *Btk*, *Lyn*, *Pira12*, *Vav3*, *Ppp3r2*
mmu04914	Progesterone-mediated oocyte maturation	10/370	0.002033	*Igf1*, *Pgr*, *Pik3cd*, *Pkmyt1*, *Mapk13*, *Pde3b*, *Rps6ka1*, *Kif22*, *Cdc25c*, *Cdc25b*

GeneRatio: ratio of differentially expressed genes annotated to the KEGG pathway to all differentially expressed genes annotated with the KEGG pathway.

**Table 4:** KEGG pathway-centric clustering analysis of differentially expressed genes in COAG vs. CG (*p*-value < 0.01).

Pathway ID	Pathway Description	GeneRatio	*p*-Value	Names of the Genes in Pathway
mmu04510	Focal adhesion	24/375	1.18 × 10^−6^	*Actg2*, *Itga4*, *Ccnd1*, *Vav1*, *Parvb*, *Igf1*, *Col6a3*, *Prkcb*, *Pdgfb*, *Rac2*, *Itga6*, *Fn1*, *Spp1*, *Thbs2*, *Pgf*, *Rasgef1b*, *Vav3*, *Pik3cd*, *Plxdc1*, *Lama5*, *Vwf*, *Itga2*, *Efs*, *Thbs3*
mmu04810	Regulation of actin cytoskeleton	25/375	4.19 × 10^−6^	*Itgal*, *Itgam*, *Itgax*, *Itgb2*, *Actg2*, *Itga4*, *Myh11*, *Vav1*, *Nckap1l*, *Cxcr4*, *Pdgfb*, *Rac2*, *Itga6*, *Lpar4*, *Fn1*, *Rasgef1b*, *Vav3*, *Pik3cd*, *Myh14*, *Myh10*, *Scin*, *Itga2*, *Fgf10*, *Efs*, *Iqgap3*
mmu04010	MAPK signaling pathway	26/375	0.000126	*Csf1r*, *Cacna1a*, *Igf1*, *Prkcb*, *Ptpn7*, *Pdgfb*, *Rac2*, *Arrb2*, *Rps6ka1*, *Pgf*, *Tgfb3*, *Tnf*, *Rasgef1b*, *Plxdc1*, *Cd14*, *Map3k7cl*, *Cacng8*, *Tgfb2*, *Arrb1*, *Tmem178*, *Hspa1b*, *Map3k8*, *Fgf10*, *Cdc25b*, *Rasgrp2*, *Mapk13*
mmu04512	ECM–receptor interaction	11/375	0.000736	*Itga4*, *Col6a3*, *Cd36*, *Itga6*, *Fn1*, *Spp1*, *Thbs2*, *Lama5*, *Vwf*, *Itga2*, *Thbs3*
mmu05205	Proteoglycans in cancer	26/375	5.86 × 10^−8^	*Actg2*, *Ptpn6*, *Plau*, *Ccnd1*, *Vav1*, *Igf1*, *Prkcb*, *Hcls1*, *Camk2a*, *Rac2*, *Fn1*, *Thbs2*, *Tnf*, *Rasgef1b*, *Vav3*, *Pik3cd)*, *Mmp2*, *Plxdc1*, *Plcg2*, *Ank3*, *Tgfb2*, *Itga2*, *Gpc3*, *Iqgap3*, *Mapk13*, *Pik3ip1*
mmu05206	MicroRNAs in cancer	19/375	1.37 × 10^−5^	*Plau*, *Ccnd1*, *Prkcb*, *Pdgfb*, *Hmga2*, *Thbs2*, *Rasgef1b*, *Mmp16*, *Pik3cd*, *Plxdc1*, *Plcg2*, *Tgfb2*, *Notch1*, *Brca1*, *Ccne2*, *Kif23*, *Cdc25b*, *Bcl2l11*, *Pik3ip1*
mmu05417	Lipid and atherosclerosis	22/375	6.95 × 10^−5^	*Ncf1*, *Abcg1*, *Vav1*, *Cd36*, *Camk2a*, *Ncf2*, *Casp1*, *Rac2*, *Ncf4*, *Lyn*, *Tnf*, *Cxcl1*, *Pycard*, *Vav3*, *Pik3cd*, *Cd14*, *Map3k7cl*, *Lbp*, *Hspa1b*, *Cxcl2*, *Bcl2l14*, *Mapk13*
mmu05134	Legionellosis	10/375	0.000129	*Itgb2*, *Casp1*, *Eef1a2*, *Naip2*, *Tnf*, *Cxcl1*, *Pycard*, *Cd14*, *Hspa1b*, *Cxcl2*
mmu05200	Pathways in cancer	39/375	0.000182	*Csf1r*, *Spi1*, *Ccnd1*, *Igf1*, *Prkcb*, *Gng2*, *Camk2a*, *Cxcr4*, *Pdgfb*, *Rac2*, *Il2rg*, *Itga6*, *Lpar4*, *Fn1*, *Csf2ra*, *Il7r*, *Csf2rb2*, *Pgf*, *Tgfb3*, *Rasgef1b*, *Pik3cd*, *Mmp2*, *Plxdc1*, *Plcg2*, *Lama5*, *Tgfb2*, *Jag1*, *Notch1*, *Itga2*, *Ccne2*, *Skp2*, *Rad51*, *Csf2rb*, *Fgf10*, *Jup*, *Rasgrp2*, *Bcl2l14*, *Bcl2l11*, *Gli2*
mmu05410	Hypertrophic cardiomyopathy	13/375	0.000191	*Actg2*, *Itga4*, *Igf1*, *Des*, *Itga6*, *Ace*, *Tgfb3*, *Tnf*, *Cacng8*, *Tgfb2*, *Itga2*, *Tmem178*, *Mybpc3*
mmu04933	AGE-RAGE signaling pathway in diabetic complications	12/375	0.000275	*Ccnd1*, *Prkcb*, *Rac2*, *Fn1*, *Tgfb3*, *Tnf*, *Pik3cd*, *Mmp2*, *Plcg2*, *Tgfb2*, *Bcl2l14*, *Mapk13*
mmu05214	Glioma	10/375	0.000739	*Ccnd1*, *Igf1*, *Prkcb*, *Camk2a*, *Pdgfb*, *Rasgef1b*, *Camk1d*, *Pik3cd*, *Plcg2*, *Bcl2l14*
mmu04380	Osteoclast differentiation	34/375	1.85 × 10^−19^	*Ncf1*, *Csf1r*, *Lilrb4a*, *Tnfrsf11a*, *Sirpa*, *Tyrobp*, *Fcgr3*, *Spi1*, *Pira2*, *Pira12*, *Milr1*, *Lcp2*, *Fcgr2b*, *Pirb*, *Lilrb4b*, *Pira1*, *Ncf2*, *Blnk*, *Btk*, *Rac2*, *Ncf4*, *Gm49339*, *Syk*, *Tnfrsf11b*, *Tnf*, *Fcgr4*, *Pik3cd*, *Plcg2*, *Map3k7cl*, *Tgfb2*, *Fosb*, *Socs3*, *Tec*, *Mapk13*
mmu04062	Chemokine signaling pathway	28/375	5.87 × 10^−10^	*Ncf1*, *Ccl9*, *Was*, *Dock2*, *Pik3cg*, *Vav1*, *Prkcb*, *Gng2*, *Cxcr4*, *Rac2*, *Ccr1*, *Arrb2*, *Prex1*, *Hck*, *Lyn*, *Cxcl1*, *Rasgef1b*, *Vav3*, *Pik3cd*, *Plcg2*, *Pik3r5*, *Grk3*, *Arrb1*, *Tec*, *Cxcl2*, *Efs*, *Rasgrp2*, *Pf4*
mmu04670	Leukocyte transendothelial migration	23/375	1.01 × 10^−9^	*Ncf1*, *Itgal*, *Itgb2*, *Actg2*, *Itga4*, *Milr1*, *Vav1*, *Prkcb*, *Ncf2*, *Cxcr4*, *Rac2*, *Ncf4*, *Cdh5*, *Vav3*, *Pik3cd*, *Mmp2*, *Plcg2*, *F11r*, *Rapgef3*, *Tec*, *Cldn4*, *Efs*, *Mapk13*
mmu04662	B cell receptor signaling pathway	26/375	2.23 × 10^−8^	*Cd72*, *Inpp5d*, *Lilrb4a*, *Ptpn6*, *Fcgr3*, *Pira2*, *Pira12*, *Milr1*, *Vav1*, *Fcgr2b*, *Prkcb*, *Pirb*, *Lilrb4b*, *Pira1*, *Pik3ap1*, *Blnk*, *Btk*, *Rac2*, *Gm49339*, *Syk*, *Lyn*, *Rasgef1b*, *Vav3*, *Pik3cd*, *Plcg2*, *Tec*
mmu04611	Platelet activation	18/375	2.59 × 10^−6^	*Fcer1g*, *Actg2*, *Pik3cg*, *Fermt3*, *Lcp2*, *Btk*, *Tbxas1*, *Syk*, *Lyn*, *Pik3cd*, *Plcg2*, *Apbb1ip*, *Pik3r5*, *Vwf*, *Itga2*, *Tec*, *Rasgrp2*, *Mapk13*
mmu04625	C-type lectin receptor signaling pathway	13/375	0.000228	*Clec7a*, *Fcer1g*, *Clec4n*, *Lsp1*, *Casp1*, *Clec4d*, *Syk*, *Tnf*, *Pycard*, *Pik3cd*, *Plcg2*, *Egr3*, *Mapk13*

GeneRatio: Ratio of differentially expressed genes annotated to the KEGG pathway to all differentially expressed genes annotated with the KEGG pathway.

**Table 5:** KEGG pathway-centric clustering analysis of differentially expressed genes in CHAG vs. COAG (*p*-value < 0.01).

Pathway ID	Pathway Description	GeneRatio	*p*-Value	Names of the Genes in Pathway
mmu04145	Phagosome	13/81	5.50 × 10^−7^	*Atp6v0d2*, *Mrc1*, *Itgb2*, *Clec7a*, *Cd36*, *Marco*, *Msr1*, *Fcgr3*, *Ncf4*, *Rac2*, *Ctss*, *Ncf2*, *Ncf1*
mmu04810	Regulation of actin cytoskeleton	7/81	0.003389	*Itgax*, *Itgb2*, *Itgam*, *Rac2*, *Nckap1l*, *Gm49368*, *Vav1*
mmu04060	Cytokine–cytokine receptor interaction	8/81	0.004224	*Ccr1*, *Il1a*, *Csf2rb2*, *Il2rg*, *Il7r*, *Il1rn*, *Tnf*, *Tnfrsf11a*
mmu05140	Leishmaniasis	10/81	1.55 × 10^−6^	*Itgb2*, *Prkcb*, *Il1a*, *Fcgr3*, *Ncf4*, *Ptpn6*, *Ncf2*, *Ncf1*, *Tnf*, *Eef1a2*
mmu05152	Tuberculosis	11/81	3.85 × 10^−5^	*Itgax*, *Atp6v0d2*, *Mrc1*, *Itgb2*, *Clec7a*, *Il1a*, *Itgam*, *Fcgr3*, *Ctss*, *Fcer1g*, *Tnf*
mmu05417	Lipid and atherosclerosis	8/81	0.000598	*Cd36*, *Casp1*, *Ncf4*, *Rac2*, *Ncf2*, *Ncf1*, *Tnf*, *Vav1*
mmu05134	Legionellosis	4/81	0.00179	*Itgb2*, *Casp1*, *Tnf*, *Eef1a2*
mmu05133	Pertussis	4/81	0.003546	*Itgb2*, *Il1a*, *Casp1*, *Tnf*
mmu05415	Diabetic cardiomyopathy	6/81	0.005367	*Prkcb*, *Cd36*, *Ncf4*, *Rac2*, *Ncf2*, *Ncf1*
mmu04930	Type II diabetes mellitus	3/81	0.006182	*Hk3*, *Tnf*, *Cacna1a*
mmu05418	Fluid shear stress and atherosclerosis	5/81	0.007561	*Il1a*, *Rac2*, *Ncf2*, *Ncf1*, *Tnf*
mmu04933	AGE-RAGE signaling pathway in diabetic complications	4/81	0.007944	*Prkcb*, *Il1a*, *Rac2*, *Tnf*
mmu04380	Osteoclast differentiation	16/81	6.69 × 10^−15^	*Pira1*, *Lilrb4a*, *Pira12*, *Il1a*, *Spi1*, *Tyrobp*, *Fcgr3*, *Ncf4*, *Lilrb4b*, *Rac2*, *Pirb*, *Gm49339*, *Ncf2*, *Ncf1*, *Tnf*, *Tnfrsf11a*
mmu04662	B cell receptor signaling pathway	12/81	5.50 × 10^−8^	*Pira1*, *Prkcb*, *Lilrb4a*, *Pira12*, *Fcgr3*, *Lilrb4b*, *Rac2*, *Inpp5d*, *Pirb*, *Gm49339*, *Ptpn6*, *Vav1*
mmu04650	Natural killer cell-mediated cytotoxicity	10/81	3.77 × 10^−5^	*Itgb2*, *Prkcb*, *Tyrobp*, *Fcgr3*, *Rac2*, *Lat2*, *Ptpn6*, *Fcer1g*, *Tnf*, *Vav1*
mmu04670	Leukocyte transendothelial migration	7/81	0.000107	*Itgb2*, *Prkcb*, *Ncf4*, *Rac2*, *Ncf2*, *Ncf1*, *Vav1*
mmu04613	Neutrophil extracellular trap formation	10/81	0.00019	*Itgb2*, *Prkcb*, *Clec7a*, *Casp1*, *Fcgr3*, *Ncf4*, *Rac2*, *Ncf2*, *Ncf1*, *Tlr7*
mmu04625	C-type lectin receptor signaling pathway	6/81	0.000253	*Clec7a*, *Casp1*, *Clec4d*, *Clec4n*, *Fcer1g*, *Tnf*
mmu04666	Fc gamma R-mediated phagocytosis	7/81	0.001101	*Prkcb*, *Fcgr3*, *Ptprc*, *Rac2*, *Inpp5d*, *Ncf1*, *Vav1*
mmu04610	Complement and coagulation cascades	4/81	0.009357	*Itgax*, *Itgb2*, *C3ar1*, *Itgam*

GeneRatio: Ratio of differentially expressed genes annotated to the KEGG pathway to all differentially expressed genes annotated with the KEGG pathway.

## Data Availability

The original contributions presented in this study are included in the article/Supplementary Materials. Further inquiries can be directed to the corresponding author(s).

## Supplementary Materials

The following supporting information can be downloaded at: https://www.mdpi.com/article/10.3390/biology14121664/s1, Table S1: Results of CHAG vs. CG differentially expressed genes; Table S2: Results of COAG vs. CG differentially expressed genes; Table S3: Results of CHAG vs. COAG differentially expressed genes; Figure S1: Morphology of SSCs in the CHAG, COAG, and CG groups at 3, 7, and 14 days of culture; Figure S2: KEGG results of CHAG vs. CG differentially expressed genes; Figure S3: GO results of CHAG vs. CG differentially expressed genes; Figure S4: KEGG results of COAG vs. CG differentially expressed genes; Figure S5: GO results of COAG vs. CG differentially expressed genes; Figure S6: KEGG results of CHAG vs. COAG differentially expressed genes; Figure S7: GO results of CHAG vs. COAG differentially expressed genes.

## Abbreviations

The following abbreviations are used in this manuscript:

AP	Alkaline phosphatase
AST	Astaxanthin
CCK8	Cell Counting Kit-8
CO	Chitosan
COS	Chitosan oligosaccharide
DMEM/F12	Dulbecco’s Modified Eagle Medium/Nutrient Mixture F-12
EDU	5-ethynyl-2′-deoxyuridine
GO	Gene Ontology
GFRα1	GDNF family receptor alpha 1
KI67	The cell proliferation antigen Ki-67
KEGG	Kyoto Encyclopedia of Genes and Genomes
MYC	Myelocytomatosis oncogene
OCT4	Octamer binding transcription factor 4
PCNA	Proliferating Cell Nuclear Antigen
PBS	Phosphate-buffered saline
P16	Cyclin-Dependent Kinase Inhibitor 2A
P21	Cyclin-Dependent Kinase Inhibitor 1A
PCR	Polymerase Chain Reaction
PLZF	Promyelocytic Leukemia Zinc Finger
Sirt1	The silent information regulator sirtuin 1
SSCs	Spermatogonial stem cells
TERT	Telomerase reverse transcriptase
VASA	DEAD-box helicase 4
