# Supplementary material for: Construction and Performance Evaluation of an Astaxanthin–Chitosan/Chitooligosaccharide Hydrogel System for Ex Vivo Culture of Murine Spermatogonial Stem Cells
Source: Biology (Basel). 2025 Nov 24;14(12):1664. doi: 10.3390/biology14121664 (PMC12729764; doi:10.3390/biology14121664)
Supplement: Supplementary file 1 [file biology-14-01664-s001.zip › Table S1 Results of CHAG vs CG differentially expressed genes.pdf]

Table S1 Results of CHAG vs CG differentially expressed genes (Top10 in Down and Up)

Gene number	P value	The expression level in CG	The expression level in CHAG	Types of differences	Gene name
ENSMUSG00000036887	3.51E-18	63.75	2.15	Down	C1qa
ENSMUSG00000027322	1.53E-17	7.26	0.26	Down	Siglec1
ENSMUSG00000042286	6.41E-15	12.21	0.95	Down	Stab1
ENSMUSG00000018927	4.90E-14	28.61	1.46	Down	Ccl6
ENSMUSG00000079419	4.64E-10	15.23	0.98	Down	Ms4a6c
ENSMUSG00000074934	7.14E-10	4.04	0.41	Down	Grem1
ENSMUSG00000040522	4.41E-09	4.74	0.45	Down	Tlr8
ENSMUSG00000059430	5.12E-09	267.96	35.99	Down	Actg2
ENSMUSG00000049130	5.93E-09	16.05	2.16	Down	C5ar1
ENSMUSG00000052160	1.70E-08	16.13	2.38	Down	Pld4
ENSMUSG00000095545	1.18E-15	0.27	5.77	Up	Zfp969
ENSMUSG00000040026	8.06E-08	36.27	224.89	Up	Saa3
novel380	9.94E-08	3.63	22.39	Up	-
ENSMUSG00000041449	8.52E-07	1.6	9.4	Up	Serpina3h
ENSMUSG00000022126	5.51E-06	1.54	7.32	Up	Acod1
ENSMUSG00000074766	1.63E-05	0.81	3.66	Up	Ism1

novel212	2.23E-05	18.03	73	Up	-
ENSMUSG00000095348	2.65E-05	0.34	4.15	Up	Gm3892
ENSMUSG00000028989	5.45E-05	1.04	4.32	Up	Angptl7
novel377	7.19E-05	6.93	25.84	Up	-
