# Supplementary material for: Construction and Performance Evaluation of an Astaxanthin–Chitosan/Chitooligosaccharide Hydrogel System for Ex Vivo Culture of Murine Spermatogonial Stem Cells
Source: Biology (Basel). 2025 Nov 24;14(12):1664. doi: 10.3390/biology14121664 (PMC12729764; doi:10.3390/biology14121664)
Supplement: Supplementary file 1 [file biology-14-01664-s001.zip › Table S2 Results of COAG vs CG differentially expressed genes.pdf]

Table S2 Results of COAG vs CG differentially expressed genes(Top10 in Down and Up)

Gene number	P value	The expression level in CG	The expression level in COAG	Types of differences	Gene name
ENSMUSG00000042286	2.65E-21	12.21	0.32	Down	Stab1
ENSMUSG00000052160	1.81E-14	16.13	0.92	Down	Pld4
ENSMUSG00000015950	2.03E-13	13.37	0.85	Down	Ncf1
ENSMUSG00000053063	7.15E-13	11.14	0.83	Down	Clec12a
ENSMUSG00000049130	3.65E-12	16.05	0.58	Down	C5ar1
ENSMUSG00000025044	3.70E-12	86.79	9.49	Down	Msr1
ENSMUSG00000030830	4.85E-12	5.78	0.44	Down	Itgal
ENSMUSG00000040613	6.88E-12	33.32	2.59	Down	Apobec1
ENSMUSG00000028459	9.91E-12	26.89	1.41	Down	Cd72
ENSMUSG00000030786	1.31E-11	12.95	1.14	Down	Itgam
ENSMUSG00000040026	2.80E-09	36.27	279.86	Up	Saa3
novel212	8.02E-06	18.03	78.85	Up	-
ENSMUSG00000041449	1.74E-05	1.6	7.37	Up	Serpina3h
ENSMUSG00000028989	6.00E-05	1.04	4.27	Up	Angptl7
ENSMUSG00000079019	0.00022	49.54	166.08	Up	Insl3

novel622	0.00022	2.33	8.14	Up	-
ENSMUSG00000074766	0.000241	0.81	2.92	Up	Ism1
ENSMUSG00000112876	0.000326	1.02	3.71	Up	Gm32443
ENSMUSG00000031169	0.000459	2.98	9.27	Up	Porcn
