# Supplementary material for: Construction and Performance Evaluation of an Astaxanthin–Chitosan/Chitooligosaccharide Hydrogel System for Ex Vivo Culture of Murine Spermatogonial Stem Cells
Source: Biology (Basel). 2025 Nov 24;14(12):1664. doi: 10.3390/biology14121664 (PMC12729764; doi:10.3390/biology14121664)
Supplement: Supplementary file 1 [file biology-14-01664-s001.zip › Table S3 Results of CHAG vs COAG differentially expressed genes.pdf]

Table S3 Results of CHAG vs COAG differentially expressed genes(Top10 in Down and Up)

Gene number	P value	The expression level in CHAG	The expression level in COAG	Types of differences	Gene name
ENSMUSG00000110195	4.45E-05	0.27	1.38	Down	Pde2a
ENSMUSG00000078898	0.002316	3.12	7.77	Down	Zfp968
ENSMUSG00000069972	0.003148	43.59	116.4	Down	Rps13-ps2
ENSMUSG00000112731	0.006134	0.45	1.29	Down	Gm47739
ENSMUSG00000040323	0.009526	3.95	9.96	Down	Gm15429
ENSMUSG00000058656	0.018357	0.14	0.35	Down	Samd12
ENSMUSG00000108845	0.018659	2.02	4.85	Down	Gm45090
ENSMUSG00000117748	0.020914	2.37	6.11	Down	Derpc
ENSMUSG00000112276	0.025538	0.65	1.2	Down	5033421B08Rik
ENSMUSG00000112449	0.025711	9.13	20.2	Down	Srp54b
ENSMUSG00000020807	1.19E-07	1.89	1.27	Up	4933427D14Rik
ENSMUSG00000079224	1.14E-05	48.73	9.67	Up	Gm6565
ENSMUSG00000030789	0.000689	4.2	1.53	Up	Itgax
ENSMUSG00000081665	0.000715	2.92	0.94	Up	Pira1
ENSMUSG00000037280	0.000868	2.14	0.65	Up	Galnt6

ENSMUSG00000029915	0.001126	4.83	1.39	Up	Clec5a
ENSMUSG00000025804	0.001233	4.17	1.35	Up	Ccr1
ENSMUSG00000028238	0.001339	11.07	3.76	Up	Atp6v0d2
ENSMUSG00000024300	0.001869	5.91	1.57	Up	Myo1f
ENSMUSG00000026712	0.001904	3.54	1.23	Up	Mrc1
