# Supplementary figures and images for: Construction and Performance Evaluation of an Astaxanthin–Chitosan/Chitooligosaccharide Hydrogel System for Ex Vivo Culture of Murine Spermatogonial Stem Cells
Source: Biology (Basel). 2025 Nov 24;14(12):1664. doi: 10.3390/biology14121664 (PMC12729764; doi:10.3390/biology14121664)

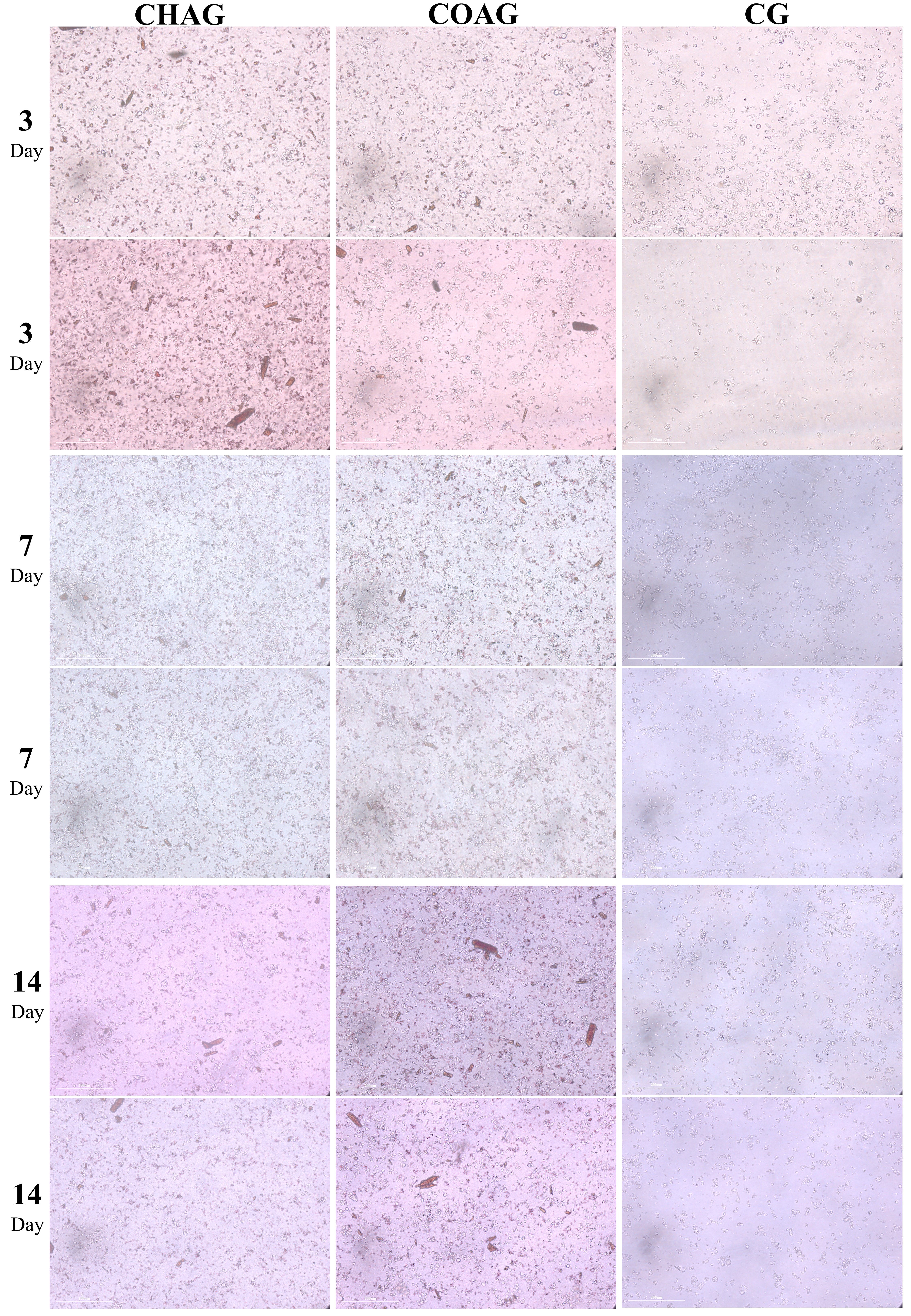

Supplement: Supplementary file 1 [file biology-14-01664-s001.zip › Figure S1 Morphology of SSCs in the CHAG, COAG and CG groups at 3, 7 and 14 days of culture.jpg]

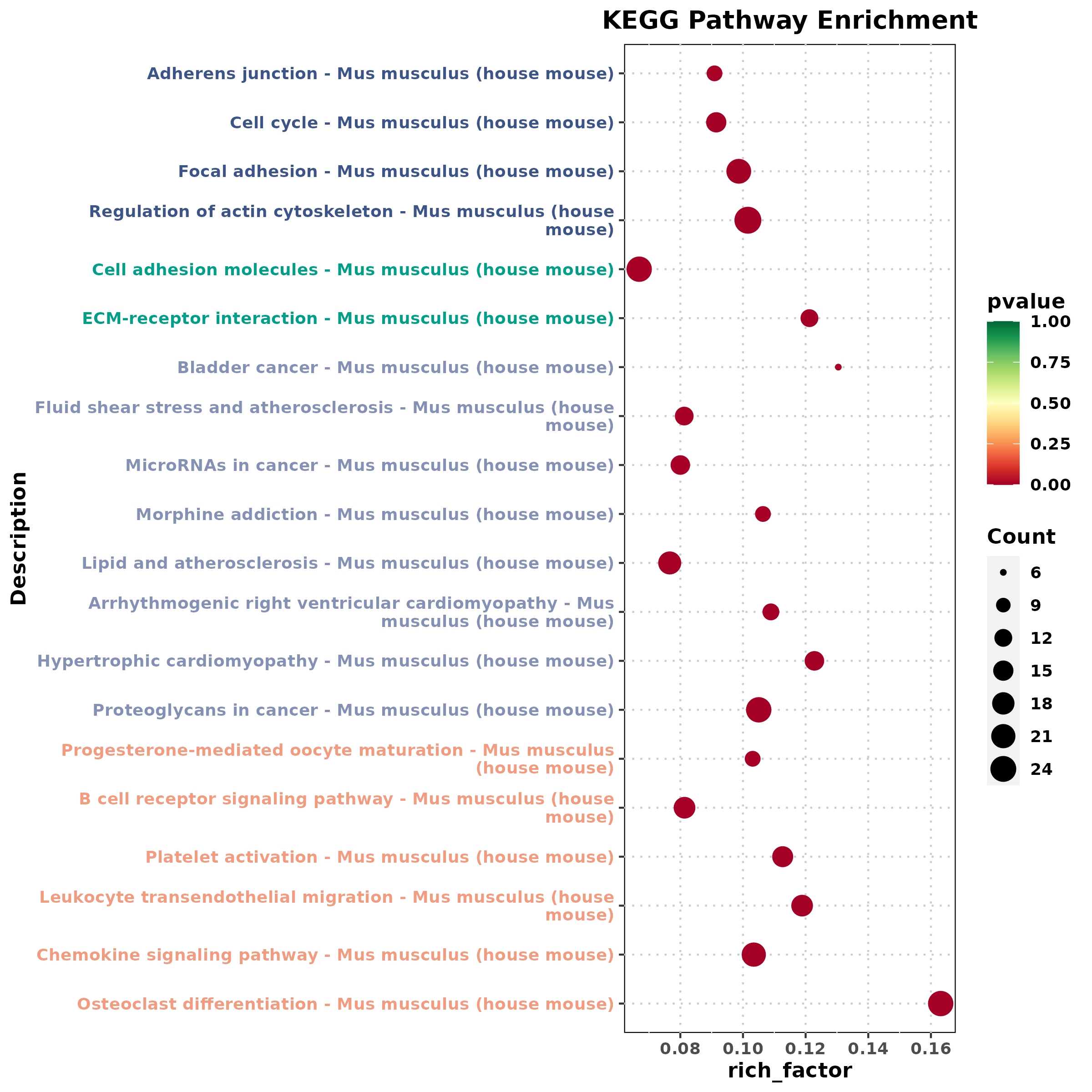

Supplement: Supplementary file 1 [file biology-14-01664-s001.zip › Figure S2 KEGG results of CHAG vs CG differentially expressed genes.jpg]

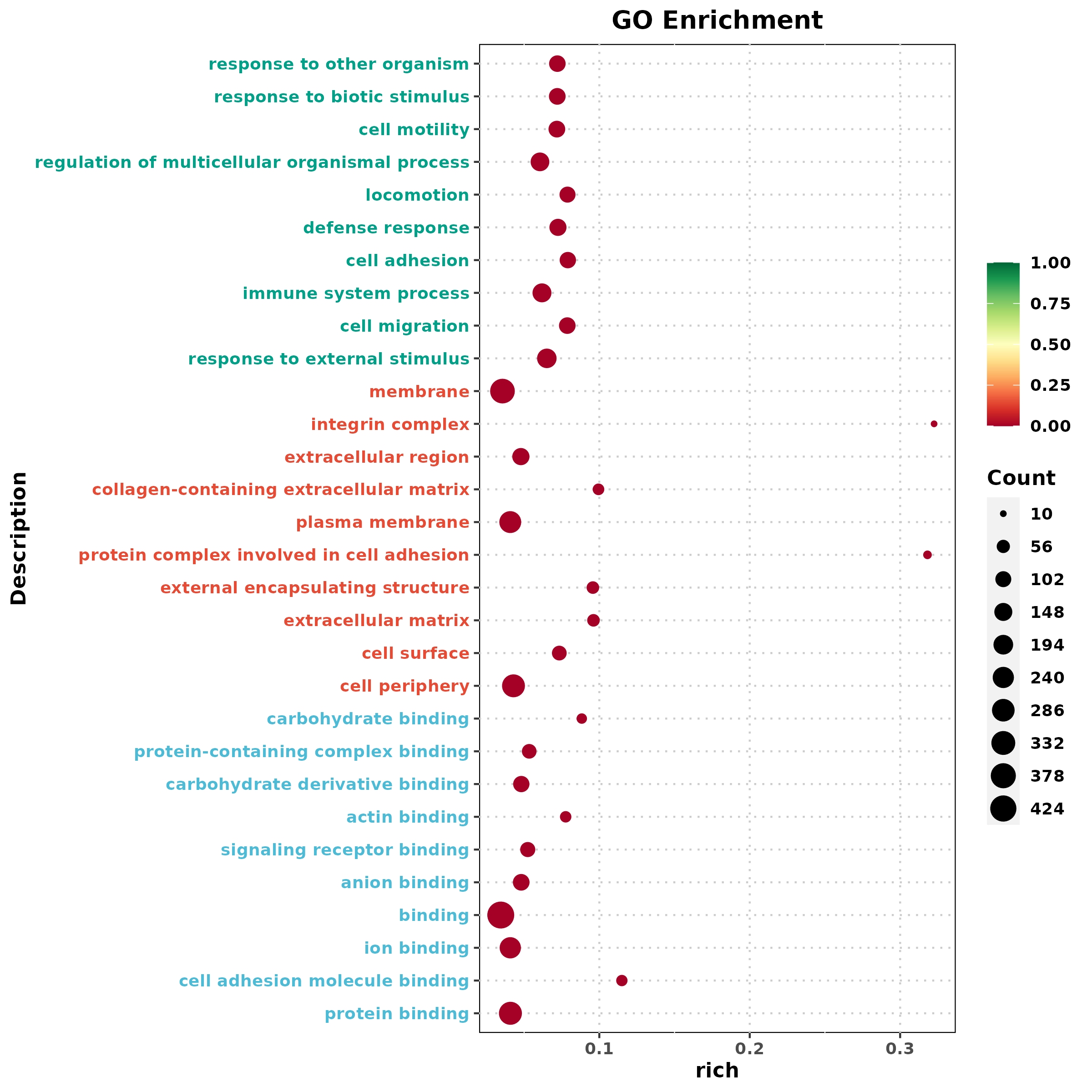

Supplement: Supplementary file 1 [file biology-14-01664-s001.zip › Figure S3 GO results of CHAG vs CG differentially expressed genes.jpg]

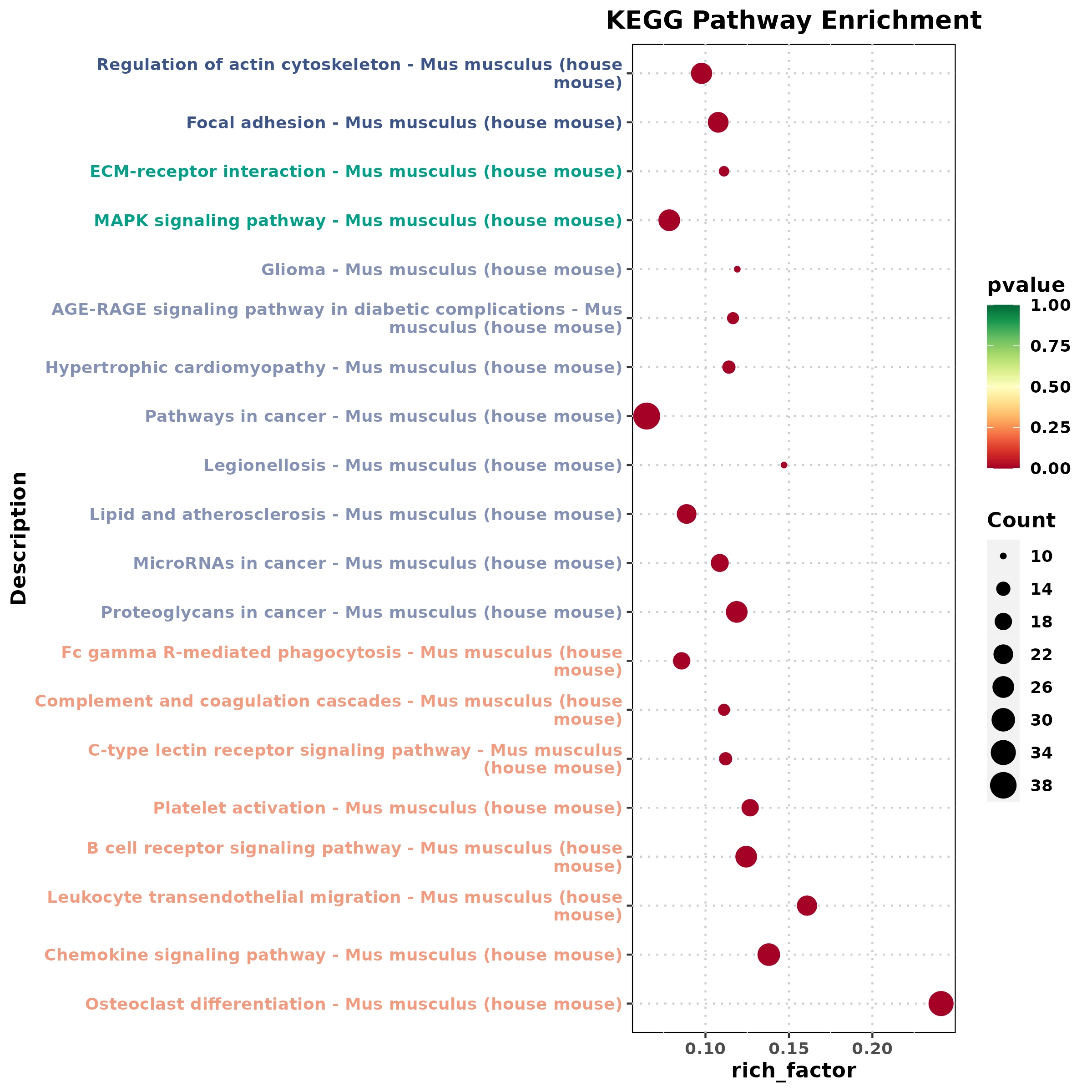

Supplement: Supplementary file 1 [file biology-14-01664-s001.zip › Figure S4 KEGG results of COAG vs CG differentially expressed genes.jpg]

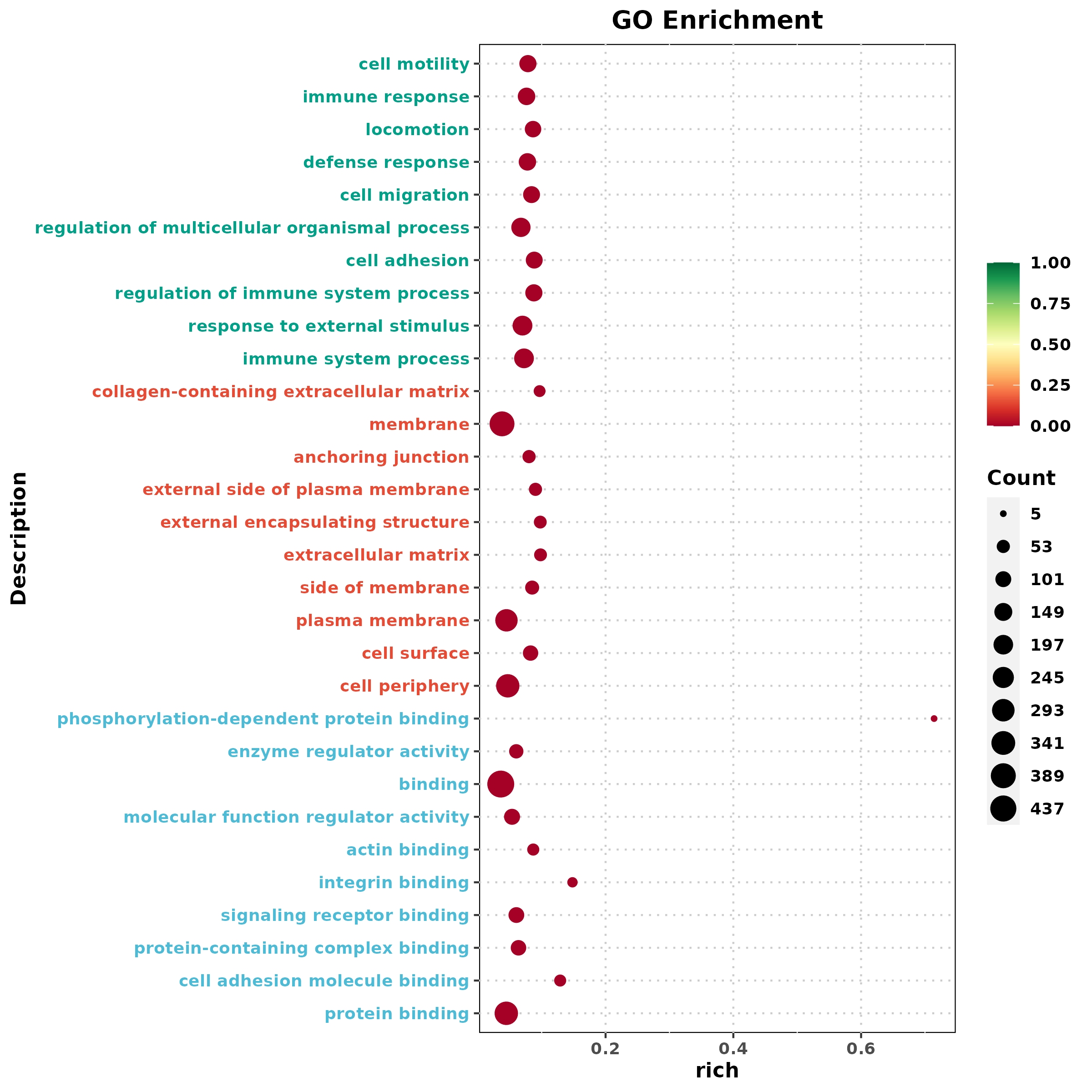

Supplement: Supplementary file 1 [file biology-14-01664-s001.zip › Figure S5 GO results of COAG vs CG differentially expressed genes.jpg]

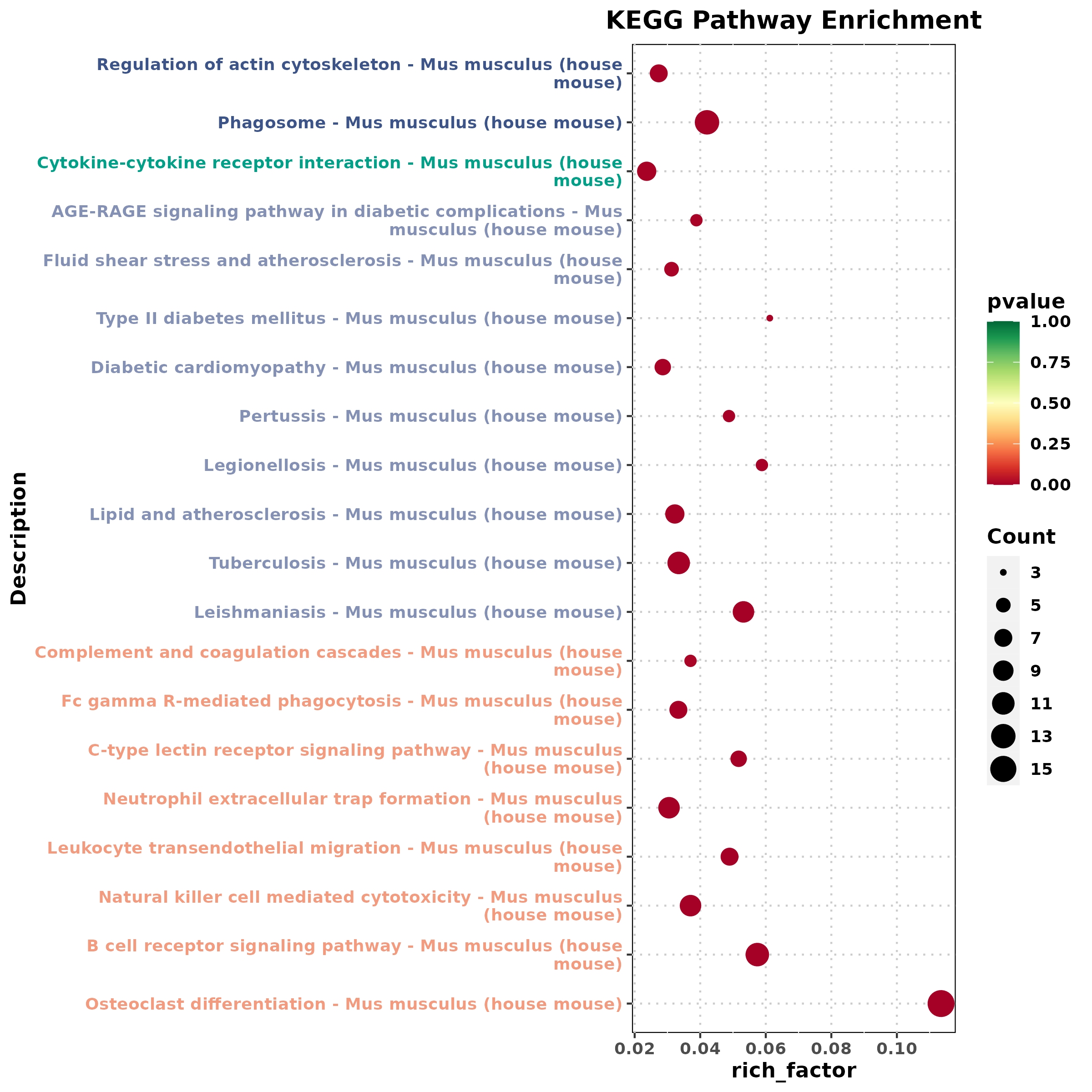

Supplement: Supplementary file 1 [file biology-14-01664-s001.zip › Figure S6 KEGG results of CHAG vs COAG differentially expressed genes.jpg]

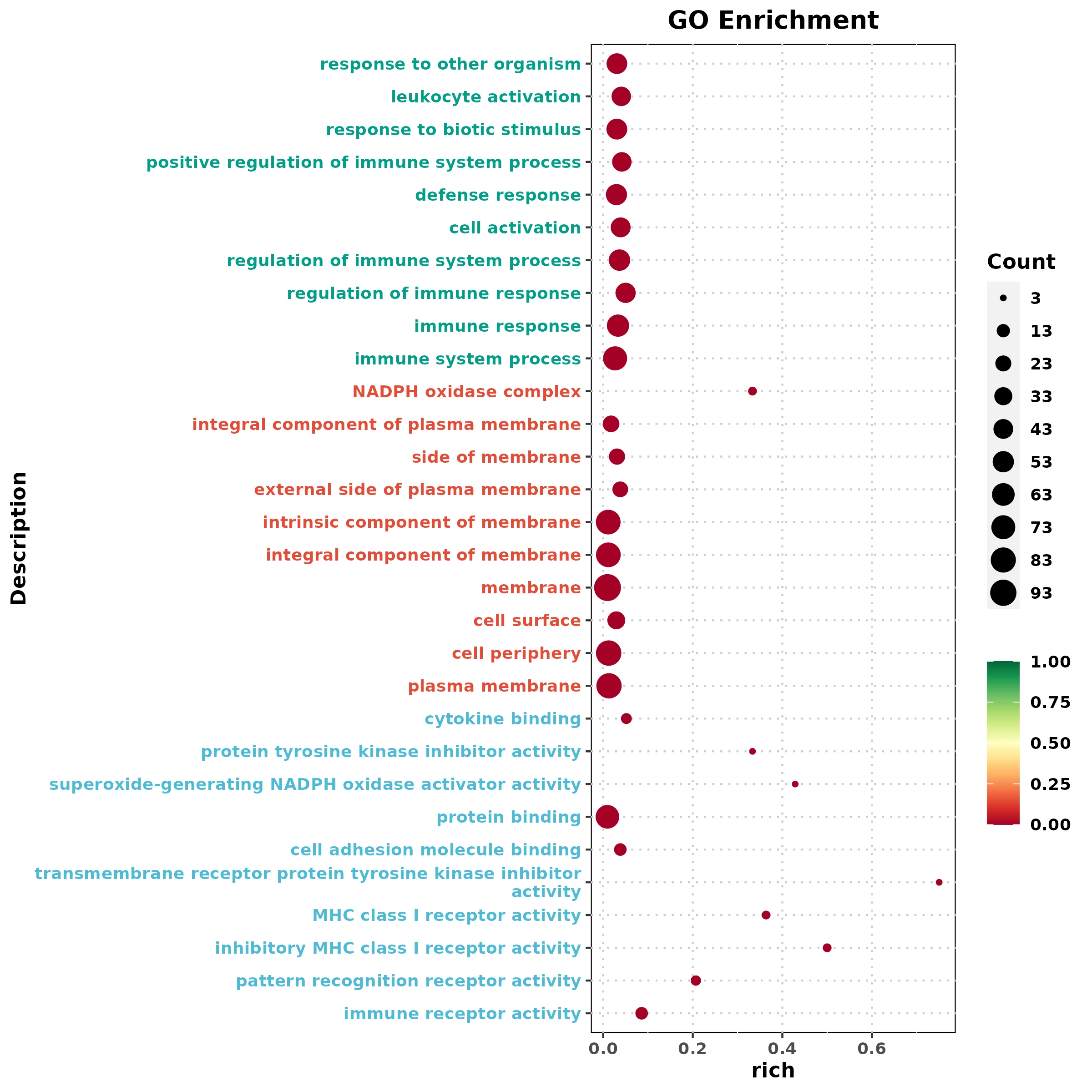

Supplement: Supplementary file 1 [file biology-14-01664-s001.zip › Figure S7 GO results of CHAG vs COAG differentially expressed genes.jpg]
